# Liposomal *Helix aspersa* Snail Mucus: A Biomacromolecular Platform for Attenuating Chronological Skin Aging Through Modulation of miR-34a-Associated Senescence and Extracellular Matrix Remodeling

**DOI:** 10.3390/ijms27177729

**Published:** 2026-08-28

**Authors:** Esraa M. Mosalam, Hend Mohamed Abdel-Bar, AbdElhafez R. AbdElhafez, Mai El-Sayed Ghoneim, Ebtehal M. Metwally, Amany Ebrahim Nofal, Aya Ibrahim Elberri

**Affiliations:** 1 Biochemistry Department, Faculty of Pharmacy, Menoufia University, Shebin EL-Kom 32511, Menoufia, Egypt; 2 Department of Pharm D, Faculty of Pharmacy, Jadara University, Irbid 21110, Jordan; 3 Leicester School of Pharmacy, Faculty of Health and Life Sciences, De Montfort University (DMU), Leicester LE19BH, UK; hend.abdelbarhammad@dmu.ac.uk; 4 Department of Pharmaceutics, Faculty of Pharmacy, University of Sadat City (USC), Sadat City 32897, Menofia Governorate, Egypt; 5 Invertebrates Division, Zoology Department, Faculty of Science, Menoufia University, Shebin El-Kom 32511, Menoufia, Egypt; hafez_rgb@science.menofia.edu.eg; 6 Department of Pharmacology and Toxicology, Faculty of Pharmacy, University of Sadat City (USC), Sadat City 32897, Menofia Governorate, Egypt; mai.ghoneim@fop.usc.edu.eg; 7 Medical Physiology Department, Faculty of Medicine, Menoufia University, Shebin El-Kom 32511, Menoufia, Egypt; ibtehal.mitwali@med.menofia.edu.eg; 8 Histology and Histochemistry Division, Zoology Department, Faculty of Science, Menoufia University, Shebin El-Kom 32511, Menoufia, Egypt; amany.nofal@science.menofia.edu.eg; 9 Genetic Engineering and Molecular Biology Division, Department of Zoology, Faculty of Science, Menoufia University, Shebin El-Kom 32511, Menoufia, Egypt; ayaelbery62@science.menofia.edu.eg

**Keywords:** *Helix aspersa*, liposomes, miR-34a-5p, Mmp1, anti-aging, ECM

## Abstract

Chronological skin aging is a complex biological process characterized by progressive collagen degradation and cellular senescence. Among the molecular regulators implicated in these hallmarks, miR-34a has emerged as a critical mediator linking inflammation and senescence pathways. Consequently, this study aimed to investigate the anti-aging potential of *Helix aspersa* snail mucus, with particular emphasis on its modulation of miR-34a-associated molecular mechanisms, and to explore liposomal encapsulation as a strategy to improve its topical performance and user acceptability. Snail mucus was collected and characterized using LC–ESI–QTOF–MS/MS, while its antioxidant activity was assessed by DPPH and FRAP assays. Afterward, mucus-loaded liposomes were developed and optimized. Aged mice were assigned to control aged, Lipo, free mucus, and Lipo-mucus groups, alongside a young control group. Anti-aging efficacy was evaluated through wrinkle grading, skin moisture determination, histological examination, and collagen assessment by Masson’s trichome staining. miR-34a-5p, its target genes, and related aging-associated genes were also determined, followed by upstream regulatory network prediction using X2Kweb. The optimized formulation exhibited nanoscale particle size, high encapsulation efficiency, enhanced skin retention, and improved spreadability. Compared with free mucus, Lipo-mucus significantly increased the epidermal and dermal deposition of total protein and allantoin. Both treatments exerted remarkable anti-aging effects, evidenced by improved skin architecture and enhanced collagen deposition. These effects were associated with modulation of the miR-34a-5p-driven senescence hub. Collectively, liposomal encapsulation enhanced the cutaneous delivery, cosmetic properties, and anti-aging efficacy of *H. aspersa* mucus, supporting its potential as a mechanism-based cosmeceutical intervention for chronological skin aging.

## 1. Introduction

Chronological skin aging is a hallmark of physiological aging characterized by progressive water loss, impaired barrier function, oxidative stress, reduced fibroblast activity, collagen degradation, wrinkle formation, dullness, and loss of skin elasticity [1]. The molecular mechanisms underlying skin aging include oxidative stress, telomere attrition, DNA damage, genetic mutations, microRNA (miRNA) dysregulation, and the accumulation of advanced glycation end-products (AGEs) [2,3,4]. Oxidative stress activates several molecular pathways such as nuclear factor-κB (NF-κB), epidermal growth factor (EGF), mitogen-activated protein kinase (MAPK), and matrix metallopeptidase (MMPs). Parallel to oxidative stress, aging is increasingly recognized as a state of chronic, low-grade inflammation, termed inflammaging, which is driven by senescent cells and their senescence-associated secretory phenotype (SASP), perpetuating cytokine production and immune dysregulation [5,6,7]. Together, oxidative stress and chronic inflammation drive structural and functional deterioration of the skin, making antioxidant and anti-inflammatory strategies attractive approaches for skin regeneration and anti-aging interventions.

A growing body of evidence has positioned microRNA-34a (miR-34a) as a molecular nexus linking oxidative stress to senescence and inflammatory pathways. Its expression is persistently elevated with age in human skin, including chronologically aged dermis and photo-exposed areas, indicating its role in both intrinsic and extrinsic skin aging [8,9]. Oxidative stress induces miR-34a via phosphoinositide 3-kinase (PI3K)- and p53-dependent pathways, while miR-34a amplifies oxidative damage by disrupting mitochondrial antioxidant systems [10,11,12]. This mechanism is closely linked to SASP, which drives persistent inflammation and extracellular matrix (ECM) degradation in aged skin [8,13,14]. Collectively, these findings identify miR-34a as a central molecular link between oxidative stress, inflammation, ECM degradation, and cellular senescence, making it an attractive therapeutic target for delaying chronological skin aging.

Snail mucus, also referred to as snail secretion filtrate or snail mucin, has gained increasing attention as a natural cosmeceutical ingredient for skin repair and rejuvenation [15]. Its biological potential is attributed to its complex composition, which includes glycoproteins, peptides, hyaluronic acid, glycolic acid, allantoin, antioxidants, and antimicrobials. These constituents contribute to skin hydration, ECM support, wound healing, fibroblast activation, collagen and elastin synthesis, and improvement in photoaging-associated changes [16]. Despite its therapeutic promise, the topical application of snail mucus remains limited by several challenges, including the lack of standardized collection procedures, batch-to-batch variability related to species, diet, and extraction conditions, limited clinical evidence, and potential irritation or hypersensitivity due to foreign proteins [17]. Moreover, its high viscosity, sticky texture, variable appearance, and possible microbial contamination may reduce consumer acceptability and long-term compliance [18]. Therefore, developing standardized and cosmetically acceptable delivery systems is essential to improve the stability, usability, and topical performance of snail mucus [15].

Liposomes represent a rational carrier platform for this purpose. As biocompatible phospholipid vesicles, liposomes can encapsulate both hydrophilic and lipophilic compounds, protect sensitive bioactives from degradation, and enhance their deposition within the skin [19,20]. Their phospholipid bilayer resembles biological membranes, enabling favorable interaction with skin lipids and improved localization of active compounds within superficial epidermal layers [21,22]. In addition, liposomes can promote local retention and provide controlled or sustained release, which may prolong the moisturizing, reparative, and regenerative effects of snail mucus [20]. Importantly, liposomal encapsulation may also improve the sensory and esthetic properties of snail mucus-based formulations [23].

By reducing direct perception of the raw mucus phase, liposomes may improve formulation homogeneity, appearance, texture, and overall consumer acceptability. Consumer acceptance is essential because successful topical anti-aging products must combine biological efficacy with ease of application and pleasant sensory properties. Accordingly, liposomal encapsulation offers a promising strategy to improve the stability, skin retention, epidermal delivery, and cosmetic acceptability of snail mucus, providing a more effective and clinically translatable anti-aging formulation than free mucus. Based on these insights, this study evaluated the anti-aging potential of *Helix aspersa* snail mucus, with particular focus on its ability to modulate the miR-34a-5p pathway associated with ECM homeostasis, cellular senescence, and collagen remodeling. The mucus was further incorporated into a liposomal delivery system to enhance topical efficacy.

## 2. Results

### 2.1. Metabolomic Profile of H. aspersa Mucus by LC–ESI–QTOF–MS/MS

**Figure 1:**
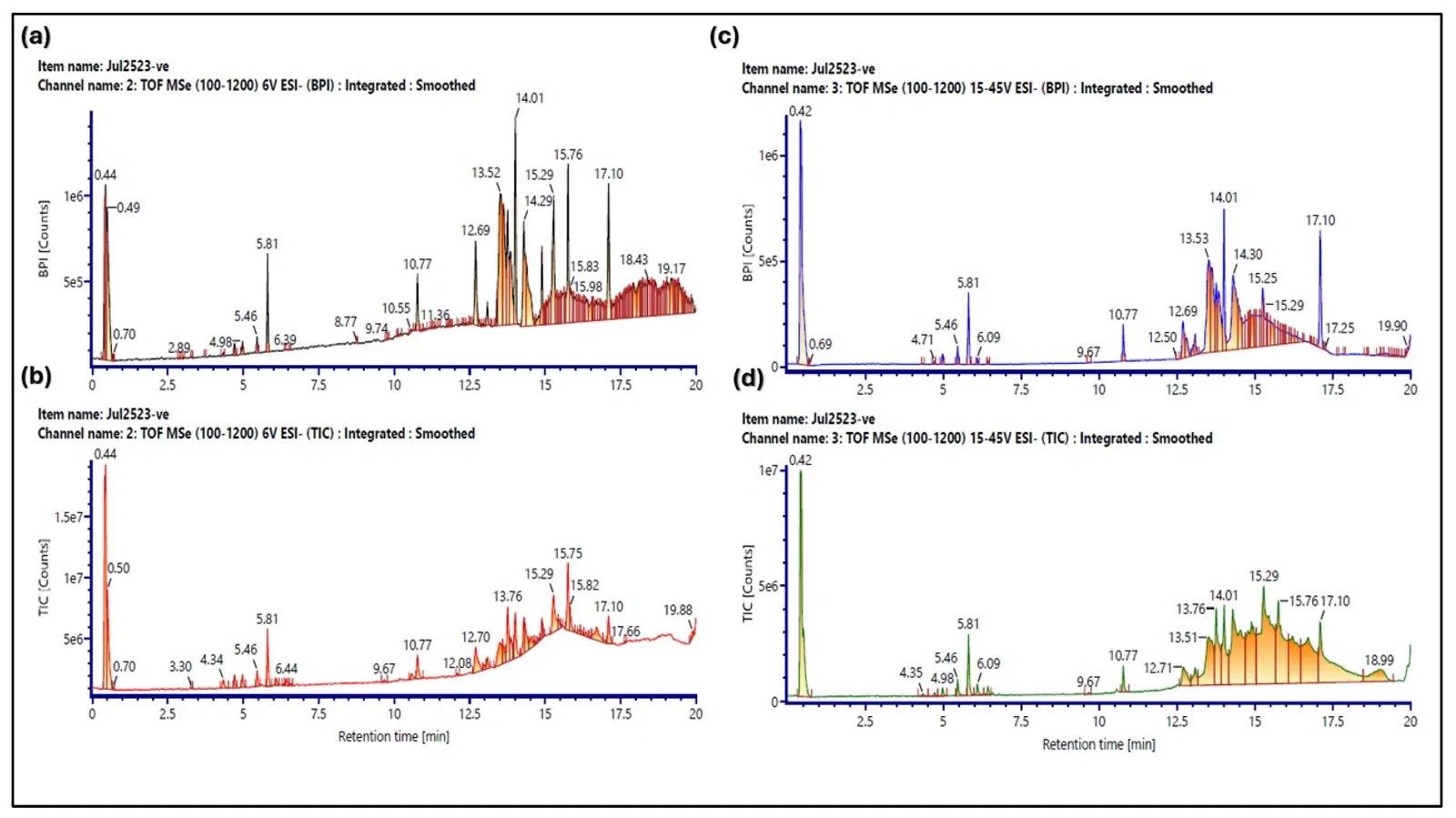
LC-ESI-QTOF-MS/MS representative chromatographic profiling of *H. aspersa* extract in negative electrospray ionization (ESI−) mode over 20 min gradient. (**a**) Base peak intensity (BPI) chromatogram recorded at low collision energy (6 V). (**b**) Total ion chromatogram (TIC) recorded at low collision energy (6 V). (**c**) BPI chromatogram recorded at higher collision energy (15–45 V). (**d**) TIC chromatogram recorded at higher collision energy (15–45 V). Major peaks are labeled with their corresponding retention times (min).

Analysis of *H. aspersa* mucus in negative ion mode revealed a highly complex chromatographic profile spanning the 20 min gradient with a total of 269 detected peaks. The low-energy (6 V, ESI−) base peak intensity chromatogram (BPI) and total ion chromatogram (TIC) displayed multiple abundant features, with particularly intense signals in the early elution region (~0.4–0.7 min) and a broad cluster of high-intensity peaks between approximately 12 and 17 min, indicating the presence of both highly polar and hydrophobic-retained metabolites within the mucus (Figure 1a,b). The corresponding high-energy (15–45 V, ESI−) BPI and TIC chromatograms exhibited similar retention patterns with richer fragmentation profiles, confirming efficient MSᴱ acquisition of fragmentation information across the chromatographic run (Figure 1c,d).

Based on retention behavior and ionization characteristics, the dominant features eluting near the void volume are consistent with highly polar small molecules, such as organic and hydroxy acids (e.g., glycolic and lactic acid derivatives) and allantoin-related metabolites, which have been largely detected in *H. aspersa* mucus and related snail extracts [24,25,26]. In addition, this early region may contain low-molecular-weight glycan-related fragments or carbohydrate derivatives, reflecting the plentiful mucins and glycosaminoglycans (GAGs) in snail mucus, although intact polymeric species are not expected to be detected under the present LC-MS conditions [27,28].

In contrast, the retained region of the chromatogram was characterized by a series of well-resolved peaks corresponding to higher-mass analytes. The recurrence of related fragment ions across multiple precursor masses suggests families of structurally related compounds, consistent with lipid-like, glycolipid, or lipopeptide-like metabolites sharing common structural backbones with variable side chains or conjugated moieties. Such lipid-rich and conjugated biomolecular profiles are in agreement with previous chemical investigations of snail extracts [28]. Features eluting in the intermediate retention window (~4–6 min) exhibited moderate hydrophobicity and complex fragmentation behavior, compatible with glycolipid-like or peptide-derived conjugates. On the other hand, metabolites eluting between approximately 10–13 min likely represent aromatic or conjugated small molecules, possibly including phenolic-related structures as suggested by prior studies, although definitive assignment cannot be made without targeted confirmation [29,30]. At later retention times (~15–20 min), lower-intensity but structurally informative features were observed, which display rich MSᴱ fragmentation patterns indicative of highly hydrophobic and structurally complex metabolites.

### 2.2. Antioxidant Capacity and Reducing Power of H. aspersa Mucus

*H. aspersa* mucus demonstrated scavenging activity against DPPH radicals, rising from 4.99% at 50 µg/mL to 74.74% at 1600 µg/mL. The determined IC_50_ value was 1069.3 µg/mL (1.07 mg/mL). The final TEAC values were calculated from concentrations of 200–1600 µg/mL, which produced consistent responses, and the mean value was estimated to be 640.36 ± 32.08 µmol of TE/g mucus. Lower concentrations (50 and 100 µg/mL) showed inhibition values below 10% and were therefore not included in the final TEAC estimation.

Similarly, the mucus exhibited antioxidant activity in a FRAP assay. TEAC values varied between 2802.86 and 497.55 µmol TE/g across the tested concentration range of 50 to 1600 µg/mL. The TEAC values corresponding to 50 µg/mL and 100 µg/mL were excluded from the final calculation because they exhibited inordinately high values, indicating increased variability and reduced reliability at these lower concentrations. Therefore, the mean antioxidant capacity was calculated using the corresponding values for the 200–1600 µg/mL concentration range, which provided a more consistent and representative estimate of ferric reducing activity of the mucus, and it was estimated to be 697.76 ± 207.45 µmol TE/g.

### 2.3. D-Optimal Design Enabled Successful Optimization of Mucus-Loaded Liposomes

**Figure 2:**
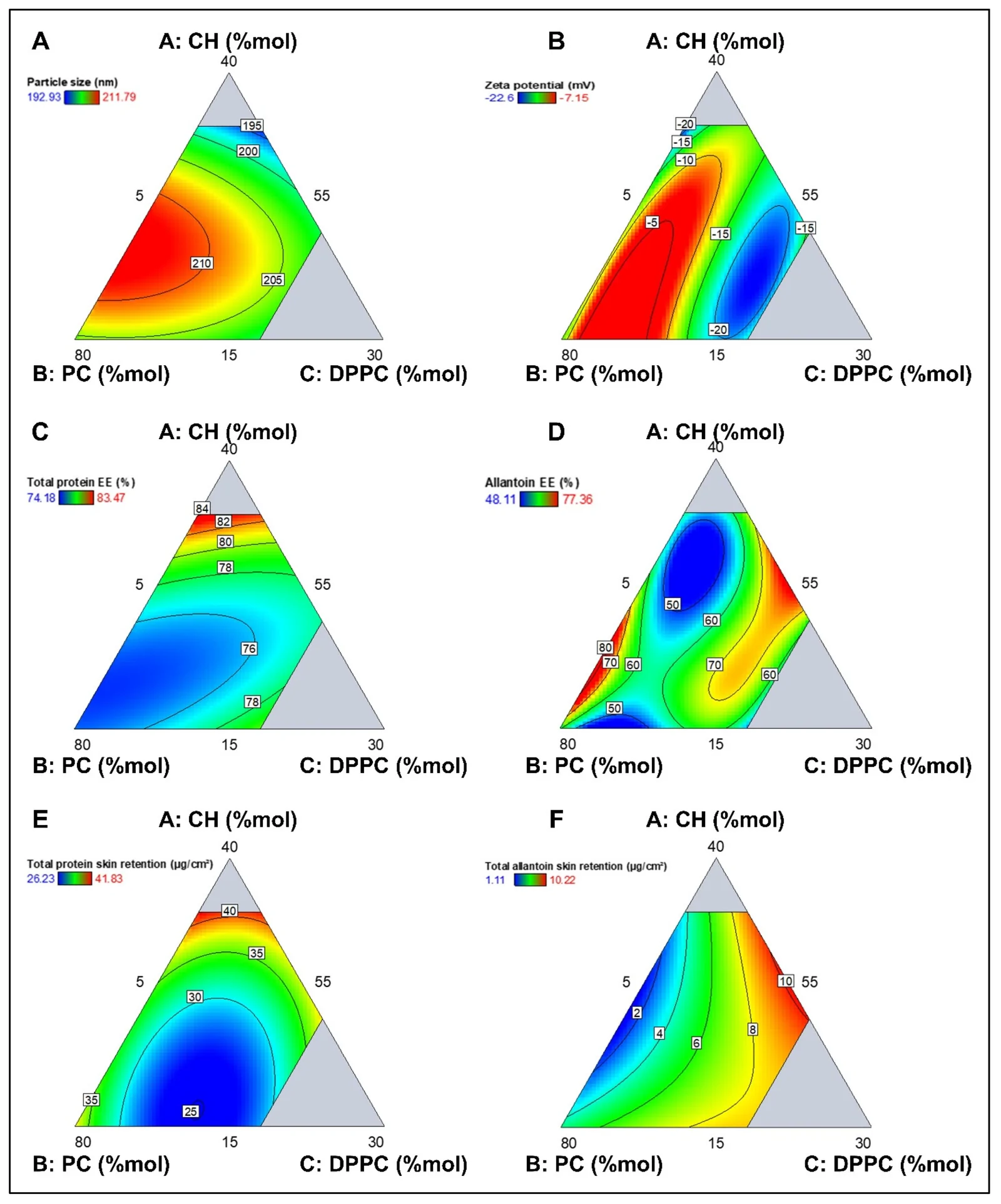
Contour plots illustrating the effects of lipid composition on the investigated responses of mucus-loaded liposomes. The plots show the model-predicted changes in (**A**) particle size, (**B**) zeta potential, (**C**) total protein encapsulation efficiency (EE %), (**D**) allantoin EE %, (**E**) total protein skin retention, and (**F**) allantoin skin retention as a function of the relative molar proportions of cholesterol (CH), phosphatidylcholine (PC), and dipalmitoylphosphatidylcholine (DPPC). The color gradient in each plot represents the predicted magnitude of the corresponding response across the experimental mixture space, while changes in the contour pattern illustrate the direction and extent of the composition-dependent effects. The plots demonstrate that the three lipid components exerted distinct and interacting effects on the investigated responses, indicating that no single lipid proportion independently optimized all formulation attributes. The response surfaces were therefore considered collectively during numerical optimization to identify the lipid composition providing the best overall balance of minimized particle size, increased absolute zeta potential, enhanced total protein and allantoin EE %, and improved skin retention.

The mucus-loaded liposomal formulations were prepared using PC, CH, and DPPC as lipid components. A D-optimal mixture design was applied by varying the molar ratios of CH, PC, and DPPC while keeping the total lipid amount and mucus concentration constant (Table S2). The design evaluated the effect of lipid composition on particle size, zeta potential, total protein EE %, allantoin EE %, total protein skin retention, and allantoin skin retention. Model summary statistics indicated that a quadratic model best fitted particle size, total protein EE %, total protein skin retention, and allantoin skin retention, whereas a cubic model was selected for zeta potential and allantoin EE % (Tables S3–S8). ANOVA further confirmed model suitability and identified the significant lipid components and interaction terms affecting each response (Tables S9–S14). The composition-dependent behavior of the formulations is visualized in the contour plots presented in Figure 2A–F. The contour gradients represent the model-predicted magnitude of each response across the investigated mixture space and therefore provide a visual indication of how changes in the relative proportions of CH, PC, and DPPC influence each formulation attribute. The contour plots were interpreted together with the fitted model coefficients to identify the principal compositional trends and to guide selection of the optimized lipid composition.

Among the investigated responses, particle size was considered a critical quality attribute because of its influence on liposomal stability, mucus loading, skin interaction, and cutaneous deposition [31]. The prepared mucus-loaded liposomes showed particle sizes ranging from 192.93 ± 10.65 to 211.79 ± 14.63 nm, confirming successful formation of nanosized vesicles across all lipid compositions (Table S2). The smallest vesicles were obtained at 35 mol% CH, 55 mol% PC, and 10 mol% DPPC, whereas the largest size was observed at 21.04 mol% CH, 73.46 mol% PC, and 5.5 mol% DPPC. Despite these differences, all formulations remained close to 200 nm, indicating good reproducibility of the preparation and extrusion process. After removal of insignificant terms, the fitted quadratic equation for particle size was:

(1) Particle size=+182.02A+204.26B+190.09C+72.26AB+58.60AC+20.15BC

Interpretation of the fitted model together with Figure 2A indicated that PC exerted the strongest size-increasing effect among the individual lipid components, while CH and DPPC also contributed positively to particle size. The interaction terms further indicated that combinations of the lipid components could increase vesicle size. Nevertheless, the relatively narrow experimental size range indicates that lipid composition modulated, rather than markedly altered, vesicle size under the applied preparation and extrusion conditions.

Zeta potential was evaluated as an indicator of vesicle surface charge and colloidal stability. All formulations showed negative zeta potential values, ranging from −7.15 ± 1.11 to −22.60 ± 2.36 mV. The fitted cubic equation for zeta potential was:

(2) $$Zeta\;potential=-33.33A-13.14B+157.24C+41.97AB-323.95AC-366.88BC\;\;\;\;\;+541.10ABC+351.79AC\left(A-C\right)+382.53BC(B-C)$$

The model and corresponding contour plot (Figure 2B) demonstrated a clearer composition-dependent effect on surface charge. Because all measured zeta potential values were negative, coefficients producing lower numerical values corresponded to a shift toward a more negative surface potential. Increasing CH and PC individually favored more negative zeta potential values, with CH showing the stronger effect, whereas increasing DPPC alone shifted the response toward less negative values. The interaction terms showed that these individual effects were not independent; in particular, the influence of DPPC changed according to the proportions of the accompanying lipids. Thus, vesicle surface charge was governed by the overall balance among CH, PC, and DPPC rather than by the concentration of a single lipid component.

Since snail mucus is a complex biological secretion rather than a single active molecule, encapsulation efficiency was assessed using two complementary markers. Total protein was used as an indirect analytical marker of the proteinaceous, mucin-containing fraction of snail mucus, rather than as a measure of biological activity or a selective marker of mucin itself, while allantoin was used as a characteristic low-molecular-weight marker [15]. Accordingly, protein-based and allantoin-based EE % were reported separately to provide a broader assessment of mucus loading within the liposomal system. Total protein EE % ranged from 74.18 ± 8.96% to 83.47 ± 12.35%, indicating efficient retention of the mucus-derived proteinaceous fraction. The highest value was obtained with the formulation containing 35 mol% CH, 55 mol% PC, and 10 mol% DPPC, whereas the lowest value was observed at 15 mol% CH, 80 mol% PC, and 5 mol% DPPC. The fitted quadratic equation was:

(3) $$Total\;protein\;EE\left(\%\right)=+90.92A+75.1B+86.79C-22.5AB-45.56AC$$

As illustrated in Figure 2C, all three lipid components contributed positively to total protein EE %, with CH showing the strongest individual effect, followed by DPPC and PC. However, some binary interactions were unfavorable, indicating that increasing bilayer packing through particular combinations of the lipid components did not necessarily result in greater association of the proteinaceous material with the vesicles. These findings indicate that effective retention of the BCA-reactive proteinaceous component required an appropriate balance among the three lipids.

Allantoin EE % ranged from 48.11 ± 3.69% to 77.36 ± 6.65%, showing a wider variation than total protein EE %, which is expected because allantoin is a smaller, more water-soluble molecule. The fitted cubic equation was:

(4) $$Allantoin\;EE\left(\%\right)=+95.85A+61.38B-299.82C+717.66AC+725.73BC-\;1163.84ABC-241.4AB\left(A-B\right)-655.64AC\left(A-C\right)-643.77BC(B-C)$$

The response surface shown in Figure 2D revealed a distinct composition-dependent pattern. CH and PC individually favored allantoin encapsulation, whereas increasing DPPC alone produced an unfavorable effect on allantoin EE %. However, the positive CH-DPPC and PC-DPPC interaction terms indicated that DPPC could support allantoin retention when present within appropriately balanced mixed bilayers. Thus, the effect of DPPC on allantoin encapsulation depended strongly on the surrounding lipid composition rather than on its individual concentration alone. This response-dependent behavior is particularly relevant for allantoin because it is a small, water-soluble constituent whose retention within the liposomal system may differ from that of proteinaceous mucus components.

Skin retention was evaluated as a key performance response because the intended anti-aging effect depends mainly on local cutaneous deposition. Total protein retention ranged from 26.23 ± 3.65 to 41.83 ± 3.65 µg/cm^2^, while allantoin retention ranged from 1.11 ± 0.31 to 10.22 ± 1.54 µg/cm^2^, confirming the influence of lipid composition on skin deposition of both mucus-derived markers. The fitted equation for total protein retention was:

(5) Total protein skin retention=+50.66A+37.7B+57.49C−38.7AB−66.27AC−85.55BC

For allantoin skin retention, the fitted equation was:

(6) Allantoin skin retention=+7.49A+4.6B+7.01C−19.65AB

The model and Figure 2D indicated that all three lipid components positively contributed to total protein skin retention, with DPPC showing the strongest individual effect, followed by CH and PC. In contrast, the negative binary interaction terms indicated that excessive combined effects of the lipid components could reduce cutaneous deposition, emphasizing the requirement for an appropriate balance between vesicle integrity and release or partitioning of the associated proteinaceous material into the skin. As illustrated in Figure 2F, CH, PC, and DPPC each positively contributed to allantoin skin retention, with CH and DPPC showing stronger individual effects than PC. However, the negative CH–PC interaction indicated that particular combinations producing increased bilayer packing could reduce the availability of allantoin for cutaneous partitioning. Collectively, these findings demonstrate that the lipid composition influenced not only encapsulation but also the subsequent cutaneous deposition of the investigated mucus-associated analytical markers.

Taken together, the fitted models and contour plots revealed distinct but interrelated roles for the three lipid components. PC primarily contributed to vesicle formation and showed the strongest individual influence on particle size. CH generally favored encapsulation of the investigated mucus-associated components and cutaneous retention while also contributing to a more negative surface charge. DPPC showed a more response-dependent behavior: its contribution was favorable for total protein association and skin retention, whereas increasing DPPC alone was less favorable for allantoin encapsulation. Importantly, several interaction terms modified, and in some cases opposed, the effects observed for individual lipid components. These findings demonstrate that formulation performance depended on balancing CH, PC, and DPPC rather than maximizing any single component. Moreover, no individual lipid composition simultaneously provided the optimum value for all six responses, supporting the use of a multi-response desirability approach for formulation selection.

Numerical optimization was then performed to identify the lipid composition that best met the predefined goals: minimized particle size, maximized absolute zeta potential, enhanced EE %, and improved skin retention. Based on the desirability function, the formulation containing 35 mol% CH, 55 mol% PC, and 10 mol% DPPC was selected as the optimized formulation, with an overall desirability of 0.780. Experimental validation showed acceptable agreement with predicted responses, with prediction errors ranging from 3.05% to 15.07% (Table S15). The optimized mucus-loaded liposomal formulation is hereafter referred to as Lipo-mucus for simplicity throughout the subsequent sections.

### 2.4. Lipo-Mucus Exhibits Favorable Stable Vesicular, Physicochemical, and Cosmetic Properties

The Lipo-mucus showed loading capacity values of 8.79 ± 2.76% and 6.25 ± 1.96% for total protein and allantoin, respectively (Table 1). These results confirm that the optimized liposomal system was able to retain a considerable fraction of both the high- and low-molecular-weight mucus marker.

**Figure 3:**
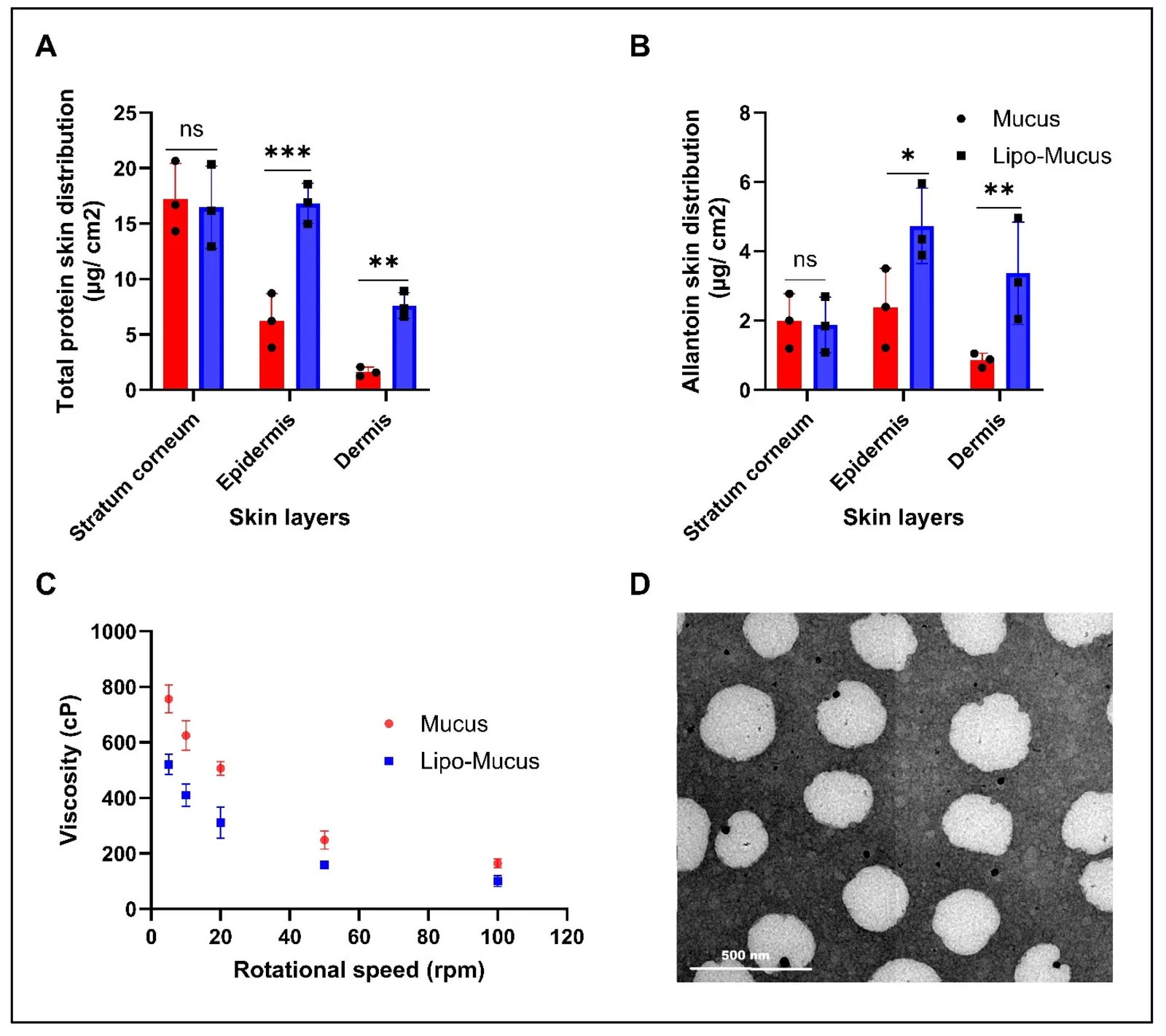
Lipo-mucus formulation enhances cutaneous deposition and improves topical characteristics compared with free mucus. Characterization of the optimized mucus-loaded liposomal formulation (Lipo-mucus) compared with free mucus. (**A**) Layer-specific distribution of total protein in the stratum corneum, epidermis, and dermis after 24 h skin application. (**B**) Layer-specific distribution of allantoin across the same skin layers. (**C**) Viscosity profiles of free mucus and Lipo-mucus measured at different rotational speeds, showing shear-thinning behavior. (**D**) TEM micrograph of Lipo-mucus showing spherical to nearly spherical vesicular structures. Data are presented as mean ± SD (*n* = 3). Statistical significance: ns, non-significant; * *p* < 0.05; ** *p* < 0.01; *** *p* < 0.001. Scale bar = 500 nm.

Layer-specific skin distribution was evaluated to determine whether the optimized formulation, Lipo-mucus, could improve the localization of mucus-derived markers within the stratum corneum, epidermis, and dermis compared with free mucus. As shown in Figure 3A, free mucus showed predominant total protein accumulation in the stratum corneum (17.22 ± 2.62 µg/cm^2^), followed by much lower deposition in the epidermis (6.26 ± 2 µg/cm^2^) and dermis (1.63 ± 0.33 µg/cm^2^). In contrast, Lipo-mucus markedly altered the distribution profile of the proteinaceous mucus fraction. Although stratum corneum retention was comparable to free mucus (16.47 ± 3.03 µg/cm^2^), epidermal and dermal deposition significantly increased to 16.83 ± 1.47 µg/cm^2^ and 7.62 ± 0.95 µg/cm^2^, respectively (*p* < 0.05).

A similar but more pronounced redistribution was observed for allantoin, as shown in Figure 3B. Free mucus produced an allantoin retention of 1.99 ± 0.65 µg/cm^2^ in the stratum corneum, 2.37 ± 0.93 µg/cm^2^ in the epidermis, and only 0.86 ± 0.16 µg/cm^2^ in the dermis. Following application of Lipo-mucus, stratum corneum retention remained comparable (1.88 ± 0.65 µg/cm^2^), whereas epidermal and dermal deposition significantly increased to 4.73 ± 0.88 µg/cm^2^ and 3.37 ± 1.2 µg/cm^2^, respectively (*p* < 0.05).

Table 1 shows that the pH of Lipo-mucus was 5.88 ± 0.98, compared with 6.89 ± 1.24 for free snail mucus. The viscosity of free mucus and Lipo-mucus was measured at different rotational speeds to evaluate their flow behavior. Both formulations showed a gradual decrease in viscosity as the rotational speed increased from 5 to 100 rpm, indicating shear-thinning behavior (Figure 3C). Free mucus exhibited higher viscosity at all tested speeds, decreasing from 756.67 ± 50.33 cP at 5 rpm to 164.67 ± 15.50 cP at 100 rpm. Similarly, Lipo-mucus showed a reduction in viscosity from 520.00 ± 36.06 cP to 100.33 ± 19.04 cP over the same speed range. At the selected comparative speed of 50 rpm, free mucus showed a viscosity of 248.33 ± 33.29 cP, whereas Lipo-mucus showed a lower viscosity of 159 ± 14.53 cP (Table 1). Moreover, Lipo-mucus showed a significantly higher spreadability than free mucus, as indicated by the shorter sliding time and higher spreadability value (Table 1). TEM analysis confirmed the vesicular morphology of Lipo-mucus. The micrograph showed spherical to nearly spherical vesicles with a relatively discrete distribution (Figure 3D), supporting successful liposome formation.

The stability of the optimized mucus-loaded liposomal formulation, Lipo-mucus, was evaluated for 90 days at 25 °C to assess its suitability for topical storage. Throughout the 3-month storage period, Lipo-mucus retained its original organoleptic characteristics, with no noticeable changes in appearance, color, or odor. No visible aggregation, precipitation, creaming, or phase separation was observed, indicating acceptable physical stability of the formulation at room temperature. As shown in Table S16, particle size showed only a moderate numerical increase from 201.65 ± 7.89 nm at day 0 to 232.32 ± 20.36 nm after 90 days, while the PDI remained within an acceptable range, changing from 0.25 ± 0.033 to 0.29 ± 0.02. One-way ANOVA showed that these changes were not statistically significant (*p* > 0.05), confirming that the vesicles maintained their nanoscale size and size distribution over the storage period. Zeta potential, total protein EE %, allantoin EE %, pH, viscosity, and spreadability also showed no significant changes over the storage period (*p* > 0.05). Total protein EE % remained above 81%, and allantoin EE % remained above 67%, indicating preservation of both mucus-derived markers. Overall, these findings confirm that Lipo-mucus maintained its physicochemical stability, marker retention, and topical performance characteristics over 3 months at room temperature.

### 2.5. Effect of Free and Liposomal H. aspersa Mucus on Age-Associated Skin Wrinkles and Hydration

**Figure 4:**
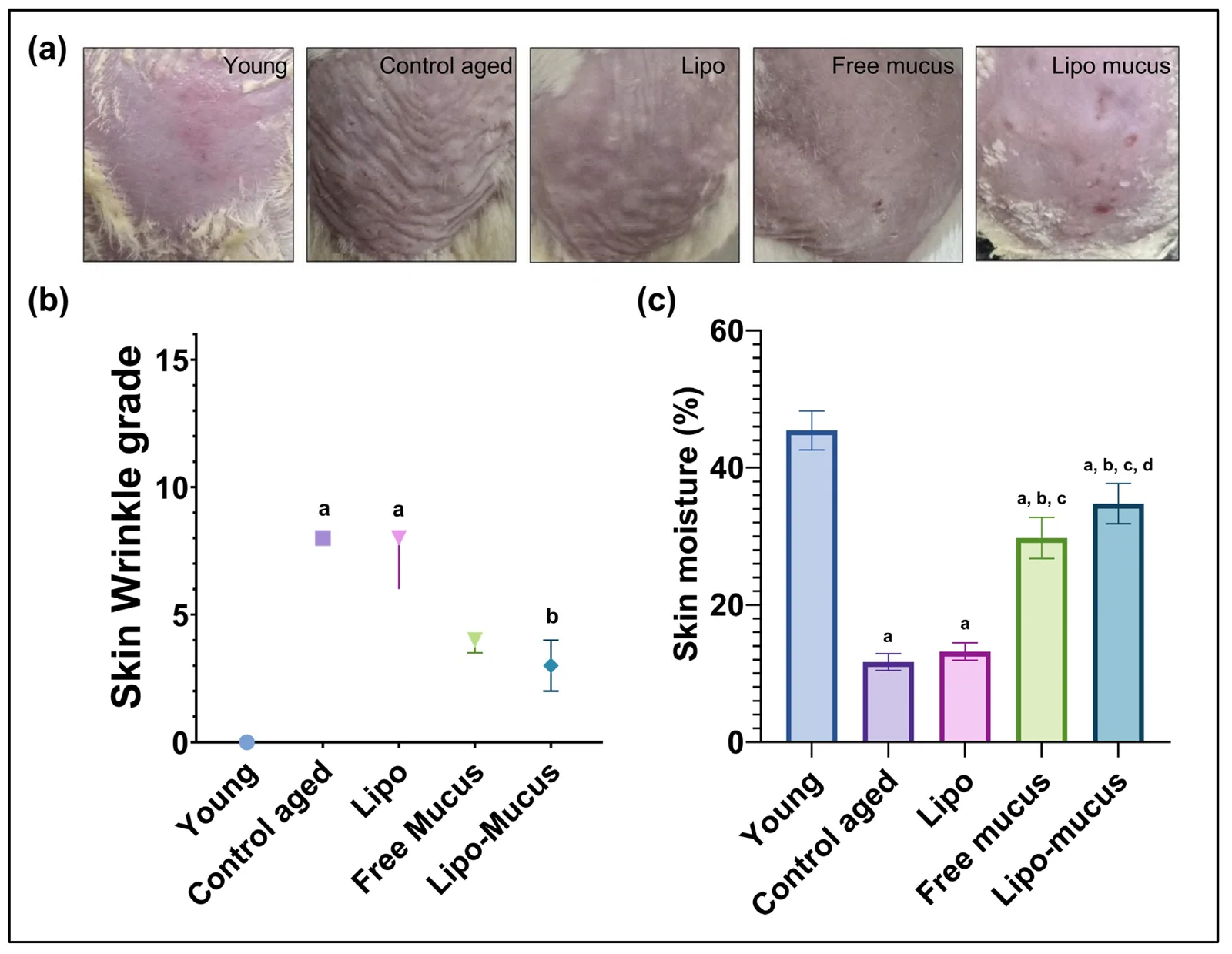
Effect of free and liposomal *H. aspersa* mucus on dorsal skin wrinkles and moisture content in mice. (**a**) Representative images of dorsal skin from the experimental mice in each studied group. (**b**) Skin wrinkle grades assessed by the predefined 0–8 ordinal scoring system (0 = no visible wrinkles and 8 = numerous deep and long wrinkles). Data are presented as medians and interquartile ranges (IQRs) for 6 mice analyzed by the Kruskal–Wallis test followed by the Dunn–Bonferroni post hoc test. (**c**) Skin moisture content (%). Data are presented as mean ± SD for 6 mice analyzed by one-way ANOVA followed by Tukey’s post hoc test. ^a,b,c,d^ *p* < 0.05 compared to young, control aged, Lipo, and free mucus group; respectively.

Wrinkle severity was assessed using the predefined wrinkle grading system (0–8). Figure 4a displays representative dorsal skin images for each studied group. The young group demonstrated no visible wrinkles, with a median score of 0 and an IQR of 0–0. In contrast, the aged group showed severe wrinkle formation with a median score of 8 and an IQR of 8–8. The Lipo group exhibited a median wrinkle grade of 8 with an IQR of 6–8, indicating severe wrinkle formation, which might be slightly less than the aged group. Treatment of the mice with free mucus reduced wrinkle severity, with a median grade of 4 and a narrow IQR of 3.5–4; indicating partial improvement but residual moderate wrinkling still present. The Lipo-mucus group showed the greatest reduction in wrinkle scores, with a median of 3 and an IQR of 2–4; demonstrating a downward shift compared with wrinkles of the aged controls.

The Kruskal–Wallis test revealed a statistically significant difference in wrinkle formation across the studied groups, as displayed in Figure 4b. Post hoc Dunn–Bonferroni comparisons confirmed significant differences between the young and aged groups as well as between young and Lipo groups (both adjusted *p* < 0.001). Notably, wrinkle scores in the Lipo-mucus group were significantly lower than those in the aged group (adjusted *p* = 0.044), whereas the reduction in the wrinkle formation observed with the free-mucus-treated mice did not remain statistically significant after adjustment by Bonferroni (*p* was 0.015 and the adjusted *p* = 0.146). No significant difference was observed between the aged and the Lipo groups.

Furthermore, Figure 4c illustrates that aging correlates with a notable decrease in skin moisture content, highlighted by a significant reduction (*p* < 0.001) of 74.32% in the control aged group relative to the young group. The topical application of the liposomal formulation without the snail mucus, Lipo, showed insignificant enhancement, demonstrating values similar to those of the control aged. In contrast, the application of free snail mucus resulted in a significant 2.55-fold increase (*p* < 0.001) in skin moisture levels compared to the aged control, suggesting a partial restoration of skin hydration. The group treated with liposomal snail mucus demonstrated a significant increase (*p* < 0.001) in moisture content by 2.98-fold compared to the control aged group.

### 2.6. Effect of Free and Liposomal H. aspersa Mucus on H&E and Masson’s Trichrome

**Figure 5:**
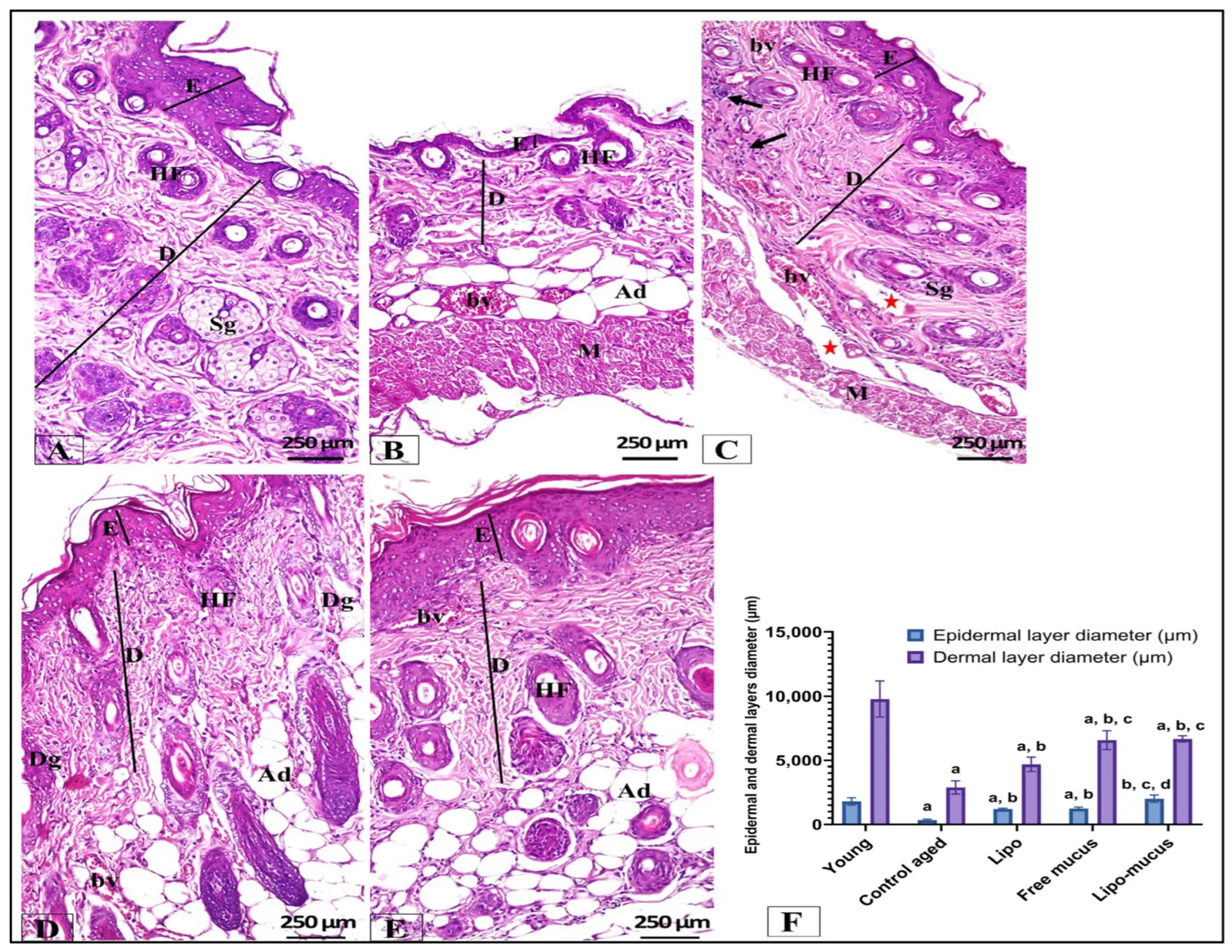
Representative photomicrographs of H&E-stained dorsal skin sections from experimental mice. (**A**) Young group, (**B**) Control aged group, (**C**) Lipo group, (**D**) free mucus group, and (**E**) Lipo-mucus group. Thin arrow, dermal and hypodermal inflammation; E, Epidermis; D, dermis; HF, hair follicle; SG, sebaceous gland; Ad, adipose tissue; BV, blood vessel; M, muscles; Dg, epidermal degeneration, Stars, odema in dermis and hypodermis layers. (**F**) Statistical analysis chart of epidermal and dermal layers thickness (μm). Data are presented as mean ± SD for 5 mice analyzed by Welch ANOVA, followed by Games-Howell test. ^a,b,c,d^ *p* < 0.05 compared to young, control aged, Lipo, and free mucus group; respectively. ×200 magnification.

Histological examination of skin sections from the young group displayed normal histological epidermal and dermal layers structure with normal extended sebaceous glands and hair follicles (Figure 5A). Control aged group exhibited a marked dermal and epidermal layers thinning, decreased dermal hair follicles, and congestion of blood vessels (Figure 5B). Lipo group showed a mild restoration of the dermal and epidermal layer thickness, increased hair follicles, but with dermal and hypodermal inflammation; few dermal inflammatory cells’ aggregations extending to the hypodermal layer with edema, and increased congested blood vessels (Figure 5C). The free mucus group showed a moderate restoration of the dermal and epidermal layer thickness, increased hair follicles with a slight epidermal degeneration and few dermal appendages (Figure 5D). The Lipo-mucus group showed a significant restoration of the dermal and epidermal layer thickness, semi-typical skin architecture with proliferated epidermal layers, semi-normal extended hair follicles, and a few congested blood vessels (Figure 5E). For morphometric measurements, Figure 5 represents the diameter of epidermal and dermal layers (μm) in skin H&E sections (*n* = 5 mice) among the current groups. Control aged group showed significant reduction (*p* < 0.05) in the diameter of epidermal and dermal layers compared to the young group. Compared with control aged mice, Lipo; free mucus; and Lipo-mucus groups showed a significant increase (*p* < 0.05) in the diameter of these layers. The best improvement was observed in the Lipo-mucus group compared to the control aged group.

**Figure 6:**
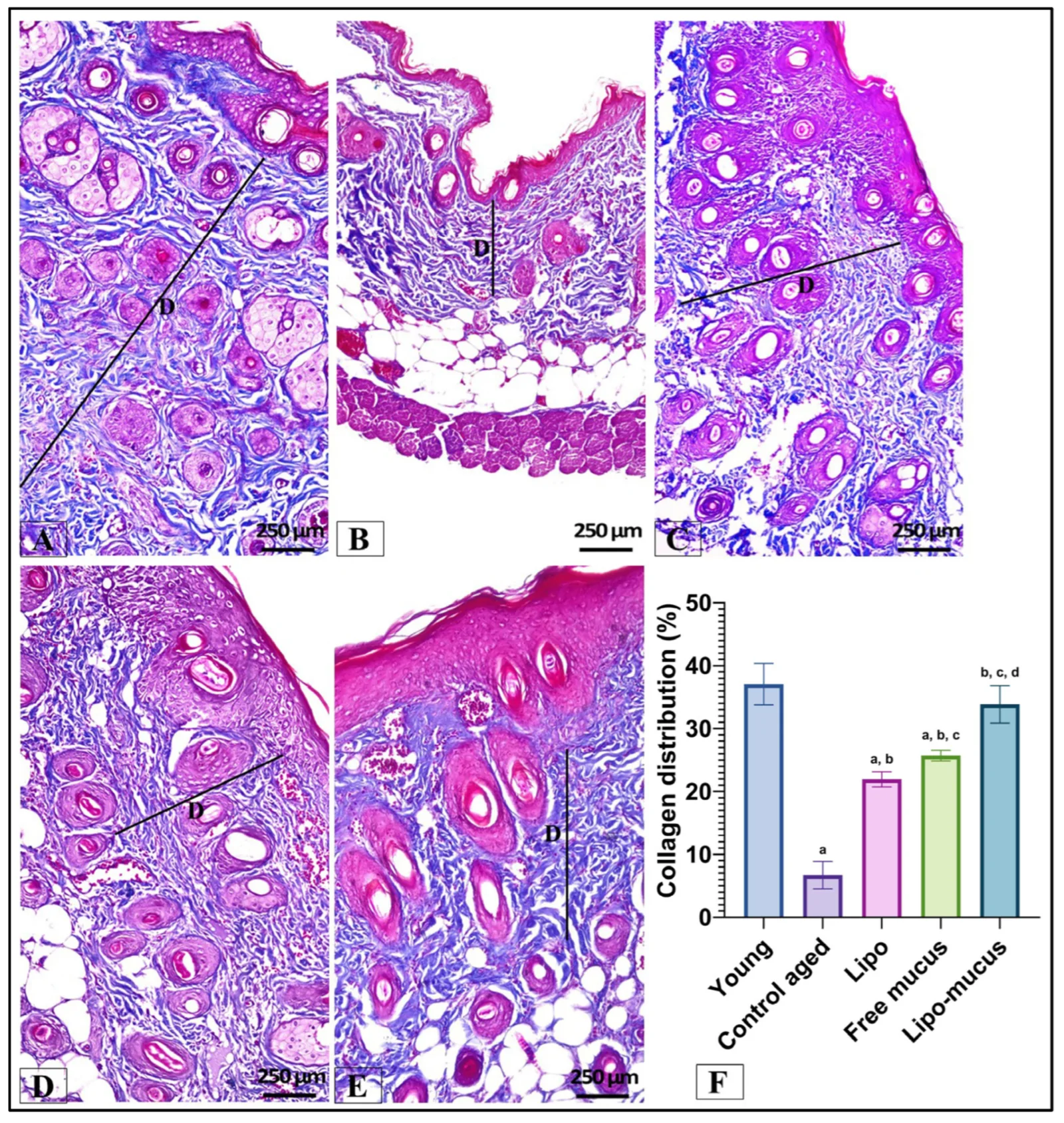
Representative photomicrographs of Masson’s trichrome-stained dorsal skin sections from experimental mice showing collagen content. (**A**) Young group, (**B**) Control aged group, (**C**) Lipo group, (**D**) free mucus group, and (**E**) Lipo-mucus group. (**F**) Statistical analysis chart of the percentage of collagen distribution. Data are presented as mean ± SD for 5 mice analyzed by Welch ANOVA, followed by Games-Howell test. ^a,b,c,d^ *p* < 0.05 compared to young, control aged, Lipo, and free mucus group; respectively. ×200 magnification.

Masson’s trichrome staining sections from young group revealed dense, uniformly distributed collagen bundles within the dermis (Figure 6A). In contrast, control aged group exhibited a marked reduction and disorganization of dermal collagen fibers (Figure 6B). However, sections from Lipo, free mucus, and Lipo-mucus groups demonstrated a significant increase in collagen fiber density (Figure 6C–E). For semi-quantitative measurements, Figure 6F represents the collagen distribution (%) in the skin sections (*n* = 5 mice) of the studied groups. The control aged group exhibited a significant reduction (*p* < 0.05) in the collagen distribution compared to the young group, while the Lipo; free mucus; and Lipo-mucus groups showed a significant increase (*p* < 0.05) in the collagen distribution relative to the control aged group. The best improvement was observed in the Lipo-mucus group. Additionally, enhanced follicular activity was evident in treated animals, as indicated by the presence of newly formed hair follicles within the dermis.

### 2.7. Upstream Regulatory Network Analysis of High-Confidence miR-34a-5p Predicted Targets

**Figure 7:**
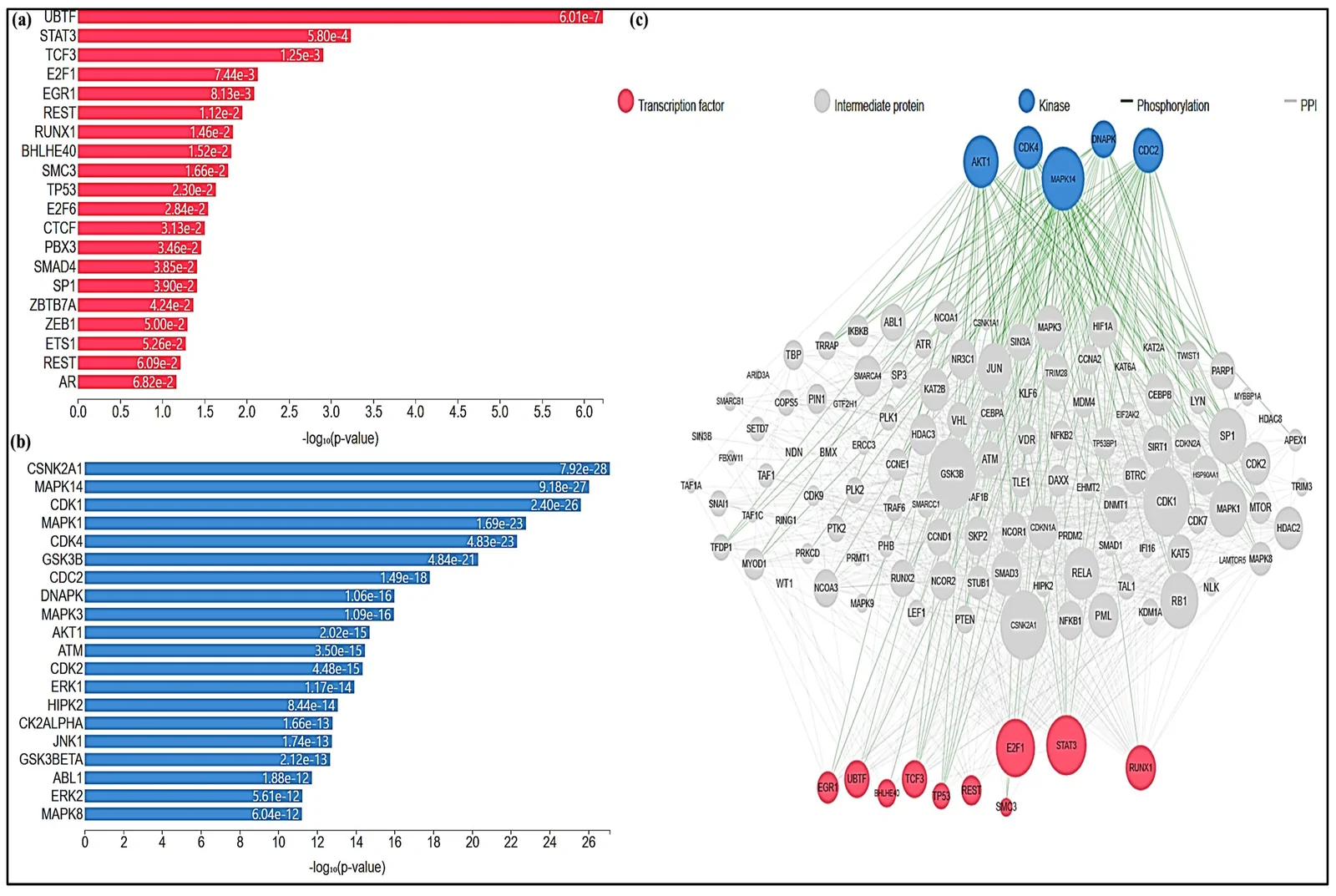
Upstream regulatory network reconstruction of high-confidence miR-34a-5p predicted targets. High-confidence predicted targets of miR-34a-5p (miRDB score ≥ 80; *n* = 244) were analyzed using X2Kweb. (**a**) Transcription Factor Enrichment Analysis (TFEA) showing the top enriched transcription factors ranked by −log10 (*p*-value); a given transcription factor may appear more than once when supported by multiple independent ChEA/ENCODE experiments, each associated with a distinct overlapping target-gene set and *p*-value. (**b**) Kinase Enrichment Analysis (KEA) showing the top enriched kinases ranked by −log10 (*p*-value). (**c**) Expression of kinases network integrating enriched transcription factors (red nodes) and kinases (blue nodes) connected *via* intermediate proteins (gray nodes); green edges represent predicted phosphorylation interactions and gray edges represent protein–protein interactions.

The 244 high-confidence predicted target genes (miRDB score ≥ 80) were subjected to upstream regulatory reconstruction using X2Kweb. Transcription factor enrichment analysis (Figure 3A) revealed significant enrichment of regulators classically implicated in cellular senescence, cell cycle control, stress signaling, and ECM regulation. Among the most significantly enriched transcription factors displayed in Figure 7a were TP53 and E2F1, which play crucial roles in cellular senescence and apoptosis. The enrichment of STAT3 indicates a potential role in inflammatory and regenerative signaling pathways. SMAD4, which has a notable connection to the aging process, was found to be enriched and serves as a key mediator of the transforming growth factor-beta (TGF-β) signaling pathway, recognized for its overactivity in various tissues during aging. The enrichment of SP1 and EGR1, which respond to oxidative stress and UV exposure, suggests a connection to transcriptional regulation associated with ECM breakdown and stress responses.

Kinase enrichment analysis (Figure 7b) further identified strong enrichment of signaling hubs associated with aging-related pathways. Multiple members of the MAPK family, including MAPK14 (p38), MAPK1 (ERK2), MAPK3 (ERK1), and MAPK8 (JNK1), were highly enriched, underscoring activation of UV- and oxidative stress-responsive signaling cascades, which are known to promote AP-1-mediated MMP expression and collagen degradation during photoaging. Enrichment of ATM and DNAPK suggests engagement of DNA damage response pathways, a hallmark of intrinsic and extrinsic skin aging. The presence of AKT1 and GSK3B further supports contribution of PI3K/AKT and Wnt-related signaling pathways that regulate epidermal homeostasis and cellular survival. Moreover, the CDK family, including CDK1/CDK2 and CDK4, supported integration with cell cycle regulation, consistent with Cdkn2a-mediated growth arrest.

The integrative X2K network analysis revealed a highly interconnected link between the enriched kinases and the downstream transcription factors through multiple intermediate signaling proteins (Figure 7c). Stress-activated kinases, principally MAPK14 (p38), AKT1, CDK4, CDC2/CDK1, and DNAPK, occupied the central upstream spots and exhibited extensive phosphorylation connections (green edges) to intermediary signaling molecules, forming a dense signaling core.

These kinases focused on major transcription factors that showed strong enrichment in the dataset, including TP53, STAT3, E2F1, RUNX1, TCF3, EGR1, and UBTF, suggesting coordinated regulation of senescence, inflammatory cascade, and cell cycle regulation. Remarkably, the network highlights an axis linking MAPK14 and ERK/JNK-related pathways to transcriptional regulators associated with UV-induced stress response and ECM remodeling. The inclusion of TP53 alongside DNA damage-related kinases (ATM/DNAPK) in the interconnected core indicates activation of genomic stress and senescence trajectories, which are defining characteristics of both intrinsic and photoinduced skin aging. Moreover, the enrichment of STAT3 and other inflammatory mediators within the PPI network suggests activation of inflammatory signaling pathways, which are highly implicated in inflammaging.

### 2.8. Alignment with Experimentally Quantified Genes

The experimentally quantified genes aligned closely with the upstream regulatory programs identified through X2Kweb analysis. Enrichment of transcription factors and kinases that are related to TP53, E2F, and CDK is consistent with the evaluation of Cdkn2a and Tert, supporting integration of the miR-34a-5p targetome within senescence and DNA damage regulatory networks. The enrichment of ATM further highlights a linkage to telomere-associated and genomic stress pathways related to aging. Likewise, the identification of multiple MAPKs family members corresponds with the assessment of ECM biomarkers like Mmp1, Plod1, and Eln, which reflect collagen degradation, matrix maturation, and elastic fiber integrity in aged skin. Enrichment of SMAD4 also aligns with the evaluation of Tgif2, supporting implication of TGF-β-associated transcriptional regulation in skin remodeling. It is worth mentioning that while the X2K analysis provides predictive upstream context rather than direct evidence of pathway activation, the concordance between enrichment analyses and experimentally quantified genes strengthens the mechanistic framework of the present study.

### 2.9. Effect of Free and Liposomal H. aspersa Mucus on miR-34a-5p and Other Genes

**Figure 8:**
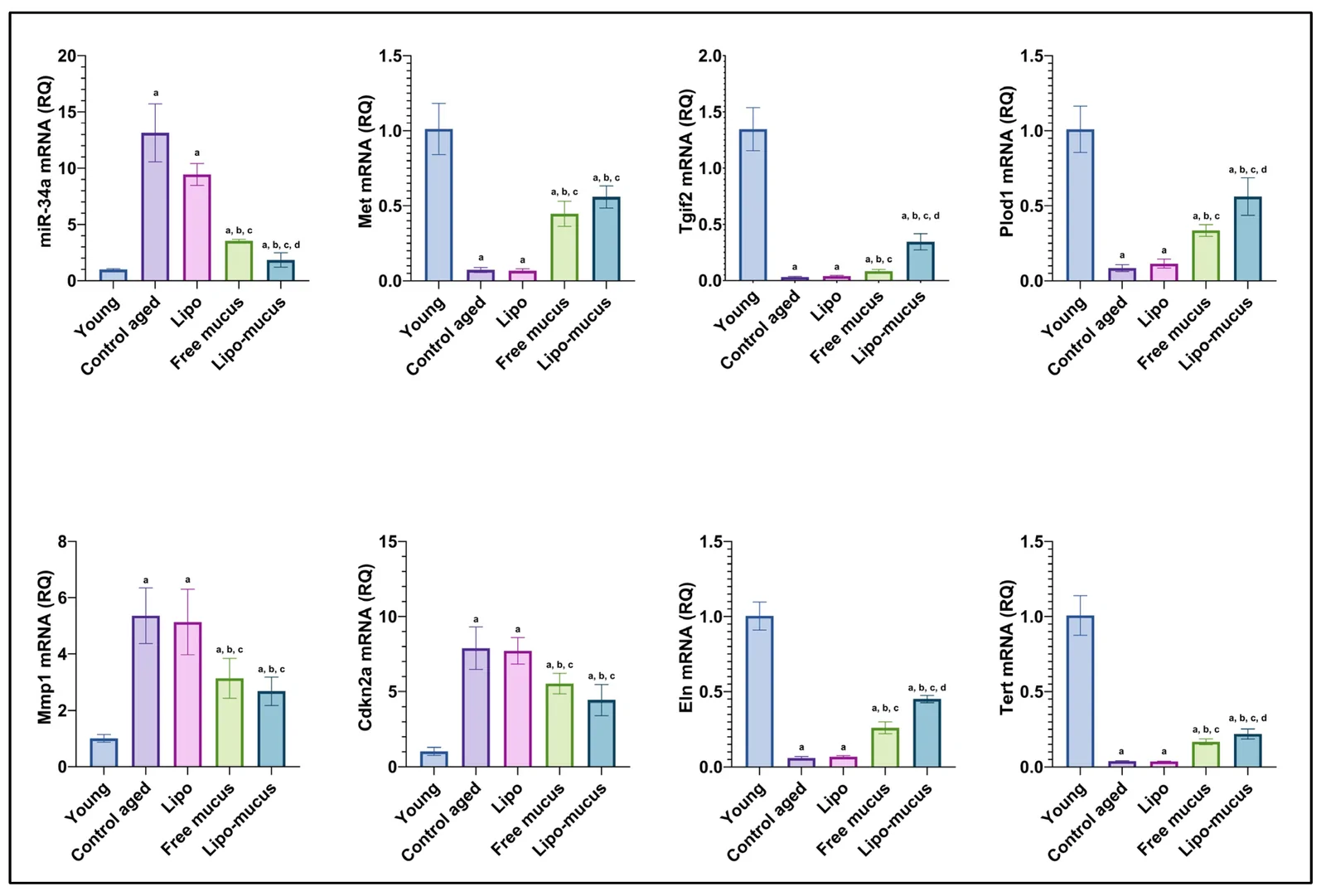
Effect of free and liposomal *H. aspersa* mucus on gene expression of miR-34a-p and other studied genes in dorsal skin from experimental mice. Data are presented as mean ± SD for 6 mice analyzed by one-way ANOVA followed by Tukey post hoc test with exception for Met; the data were analyzed by Welch ANOVA followed by Games-Howell post hoc test. ^a,b,c,d^ *p* < 0.05 compared to young, control aged, Lipo, and free mucus group; respectively. Met: met proto-oncogene, Tgif2: TGFB-induced factor homeobox 2, Plod1: procollagen-lysine, 2-oxoglutarate 5-dioxygenase 1, Mmp1: matrix metallopeptidase, Cdkn2a: cyclin-dependent kinase inhibitor 2A, Eln: elastin, Tert: telomerase reverse transcriptase.

As illustrated in Figure 8, aging significantly disrupted the miR-34a-5p axis and its related gene network. The pro-senescence miR-34a-5p was a significant (*p* < 0.001) 13.31-fold higher in control aged mice than that observed in the young controls. Treatment with free mucus resulted in a 73.07% significant (*p* < 0.001) reduction in expression, while liposomal mucus demonstrated a more significant (*p* < 0.001) downregulation of 85.97% in comparison to control aged mice.

In alignment with these observations, significant genes associated with regeneration and signaling exhibited notable alterations due to aging. In aged skin, Met, a receptor tyrosine kinase that promotes cell survival, and Tgif2, a transcriptional repressor of TGF-β signaling, exhibited significant (*p* < 0.001) suppression levels of 92.83% and 97.74%, respectively compared to young skin. The application of free mucus resulted in a partial restoration of their expression, with increases of 6.16- and 2.74-fold (*p* < 0.001), whereas the use of liposomal mucus led to an even more significant recovery, showing increases of 7.70- and 11.34-fold (*p* < 0.001) higher than the control aged mice.

In a similar manner, Plod1, which plays a crucial role in collagen cross-linking and the stabilization of the ECM, showed a significant (*p* < 0.001) reduction of 91.58% in aged mice compared to their younger counterparts. However, there was a notable increase of 3.94-fold (*p* < 0.001) by the application of free mucus and a 6.60-fold (*p* < 0.001) with the liposomal mucus formulation higher than that observed in control aged skin.

In contrast, aging increased the expression of the collagen-degrading enzyme Mmp1 by 5.31-fold (*p* < 0.001) and the senescence marker Cdkn2a by 7.68-fold (*p* < 0.001) compared to young controls. The application of free mucus resulted in a reduction in this upregulation by 41.55% (*p* = 0.001) and 29.91% (*p* = 0.025), respectively, while liposomal mucus demonstrated a more pronounced suppression of 50.04% and 43.71% (*p* < 0.001).

Moreover, Eln, responsible for encoding elastin, and Tert, the catalytic subunit of telomerase, exhibited a significant (*p* < 0.001) downregulation of 93.99% and 96.16% in aged skin compared to young skin. The application of free mucus resulted in a restoration of their expression significantly (*p* < 0.001) by 4.33- and 4.34-fold, respectively, while the application of liposomal mucus further amplified this effect in a significant manner (*p* < 0.001) to 7.48- and 5.69-fold higher than the control aged skin.

Notably, the Lipo-treated skin exhibited no significant impact and was nearly similar to the control aged akin across all measured markers. Collectively, the findings indicate that mucus, especially in its liposomal variant, significantly inhibits pro-senescence miR-34a-5p and rejuvenates essential genes related to tissue regeneration and ECM upkeep, underscoring its promise as a powerful anti-aging strategy.

### 2.10. Effect of Free and Liposomal H. aspersa Mucus on Protein Expression of VEGFA and VIM

**Figure 9:**
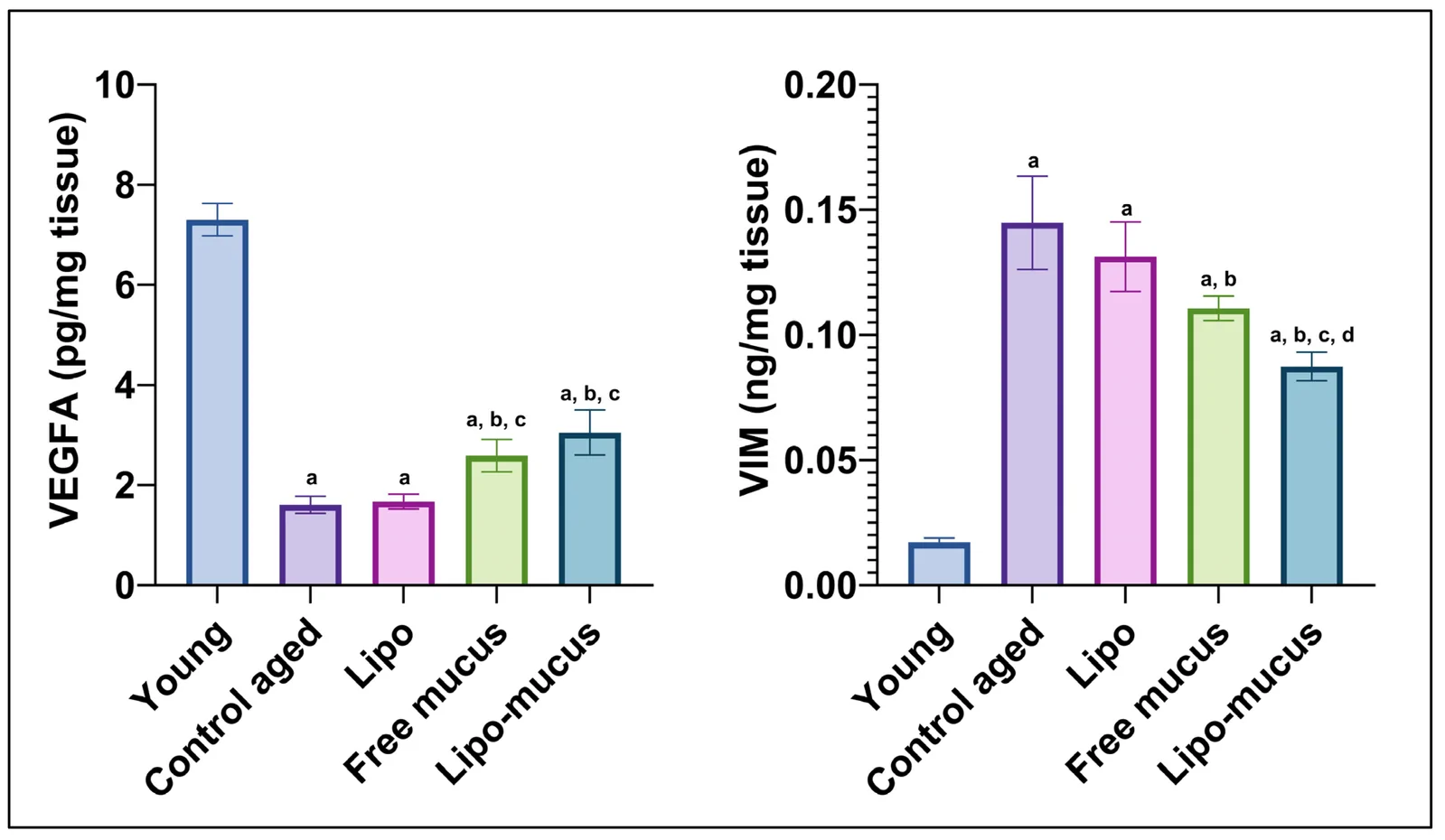
Effect of free and liposomal *H. aspersa* mucus on VEGFA and VIM in dorsal skin from experimental mice. Data are presented as mean ± SD for 6 mice analyzed by one-way ANOVA followed by Tukey post hoc test for VEGFA and by Welch ANOVA followed by Games-Howell post hoc test for VIM. ^a,b,c,d^ *p* < 0.05 compared to young, control aged, Lipo, and free mucus group; respectively. VEGFA: vascular endothelial growth factor A, VIM: vimentin.

As displayed in Figure 9, the protein levels of VEGFA and VIM were profoundly altered with aging and modulated by mucus treatments. In control aged mice, there was a significant (*p* < 0.001) reduction of 77.99% in VEGFA when compared to young controls. However, application of free mucus significantly (*p* < 0.001) restored VEGFA levels by 61.05%, whereas liposomal mucus restored it significantly (*p* < 0.001) by 89.8%, in comparison to aged mice.

In a comparable manner, VIM, a key cytoskeletal protein involved in mesenchymal integrity, was substantially elevated in aged mice by 8.47-fold (*p* < 0.001) relative to young controls. Nevertheless, application of free mucus significantly (*p* = 0.029) decreased VIM expression by 23.60%, while liposomal mucus further decreased its level (39.66%, *p* < 0.002) compared to the control aged mice. The Lipo group exhibited insignificant impact on either VEGFA or VIM, remaining similar to the aged controls. Collectively, these data suggest that mucus, especially in its liposomal form, plays a significant role in restoring angiogenic signaling and maintaining cytoskeletal balance that aging disrupts, underscoring its promise as a regenerative therapy.

## 3. Discussion

This study provides the first comprehensive mechanistic evaluation of liposomal *H. aspersa* mucus by integrating metabolomic profiling, biochemical assays, in vivo anti-aging assessment, and molecular pathway analyses.

The LC–ESI–QTOF–MS/MS analysis revealed a chemically diverse metabolomic profile in the snail mucus, encompassing both highly polar and hydrophobic compound classes. This compositional diversity aligns with previous NMR- and MS-based studies of *H. aspersa* and related gastropod species [25,32,33]. The early-eluting features including organic acids, hydroxy acids, and allantoin-related derivatives align with the established compositional data for *H. aspersa* mucus [25,34,35]. These compounds are well recognized for their keratolytic, moisturizing, and cytoprotective properties [15,36]. On the other hand, the later-eluting features exhibited higher-mass features with intricate fragmentation pattern indicative of glycolipids, lipopeptides, and phenolic conjugates. These compound classes are linked to membrane stabilization, anti-inflammatory signaling, and antioxidant activity [37,38,39,40], supporting previous chemical characterizations of snail mucus [25,33]. The hydrophilic fraction acts rapidly on the surface, inducing effects such as hydration and keratolysis, whereas the lipophilic fraction targets deeper cellular mechanisms that contribute to inflammation and oxidative stress modulation. This is a well-known multi-target activity profile in the snail mucus literature [25,32,41].

Along with the chemical complexity shown by the metabolomic profile, the *H. aspersa* mucus demonstrated considerable antioxidant capacity, as evidenced by DPPH radical scavenging and FRAP assays, thereby affirming its potential as a natural antioxidant [25,42,43,44]. These findings support previous reports indicated that *H. aspersa* mucus exhibit significant antioxidant and enzyme inhibitory activities pertinent to skin protection and anti-aging applications [45], which may elucidate the advantageous effects of snail mucus in skin protection, anti-inflammatory responses, and tissue repair [30,46]. Overall, the metabolomic complexity and antioxidant activity support the therapeutic potential of *H. aspersa* mucus for skin protection and anti-aging applications.

Wrinkle formation and skin dryness are key features of skin aging, which are driven by progressive degradation of the ECM, increased activity of MMPs, reduced collagen production, and impaired hydration [47]. Therefore, the marked wrinkling and moisture loss observed in the control aged group confirms the validity of the experimental model. Conversely, topical application of free snail mucus produced a partial but meaningful reduction in wrinkle severity and a notable improvement in skin moisture content relative to aged controls. These findings align with prior studies of dermatological activities of *H. aspersa* constituents, including the keratolytic action of glycolic acid, the humectant properties of GAGs and allantoin, and the collagen-supportive effects of mucin-associated glycoproteins [36,48]. Furthermore, these findings are consistent with prior in vivo evidence demonstrating that administration of snail mucin suppresses MMP-1/-13 expression and restores dermal collagen content in mouse skin [49]. However, the incomplete therapeutic response observed with the free mucus likely reflects intrinsic limitations in transdermal delivery since hydrophilic and high-molecular-weight macromolecules exhibit restricted permeation across the stratum corneum barrier [50,51]. Therefore, liposomal snail mucus could be a promising way to improve the efficacy and acceptability of snail mucus-based anti-aging products.

PC, CH, and DPPC were selected to formulate snail mucus-loaded liposomes with balanced vesicle-forming ability, bilayer stability, and topical suitability. PC was used as the main structural phospholipid owing to its amphiphilic nature, biocompatibility, and ability to self-assemble into liposomal vesicles [52]. CH was incorporated to modulate bilayer fluidity, enhance lipid packing, and reduce leakage of hydrophilic mucus-derived components [53]. DPPC was included as a saturated phospholipid to reinforce the bilayer and improve vesicle integrity [54]. Therefore, this lipid combination was expected to produce stable, skin-compatible liposomes capable of protecting snail mucus bioactives, supporting controlled release, and improving cutaneous deposition.

The particle-size model suggest that PC had the strongest size-increasing effect among the individual components, probably due to its dominant role as the main bilayer-forming lipid and its ability to form more hydrated and flexible vesicular structures [55]. CH and DPPC also contributed to size increase, which may be related to enhanced bilayer packing and membrane rigidity. CH can insert between phospholipid chains and regulate bilayer order, whereas DPPC, owing to its saturated acyl chains, can strengthen the vesicular membrane [56]. This increased structural ordering may reduce membrane flexibility during extrusion, resulting in slightly larger vesicles. All interaction terms were positive, indicating that combined lipid effects tended to increase particle size, particularly the CH-PC interaction (AB), followed by CH-DPPC (AC) and PC-DPPC (BC). This may be attributed to the formation of more densely packed and less deformable bilayers when these lipids were combined. The negative zeta potential values may be attributed to the association of anionic mucus-derived components, including GAGs and glycoproteins, with the vesicle surface, while the lipid components themselves are mainly zwitterionic or neutral [57]. Because all measured zeta potential values were negative, negative coefficients indicate a shift toward more negative values, whereas positive coefficients indicate a shift toward less negative values. Accordingly, the negative coefficients of CH and PC suggest that increasing these components individually favored a more negative surface charge, with CH showing the stronger effect. This may reflect the role of CH in altering bilayer packing and promoting the surface presentation of mucus-associated anionic groups, while PC showed a weaker effect due to its zwitterionic nature [58]. In contrast, the positive coefficient of DPPC suggests that increasing DPPC alone shifted the zeta potential toward less negative values, possibly by producing a more ordered bilayer that reduced the exposure or mobility of negatively charged mucus components [59]. The interaction terms revealed a more complex composition-dependent behavior. The positive CH-PC interaction shifted the zeta potential toward less negative values, whereas the negative CH-DPPC and PC-DPPC interactions promoted more negative surface charge. This indicates that the effect of DPPC depended strongly on the surrounding lipid environment; although DPPC alone reduced the magnitude of the negative charge, its combination with CH or PC may have favored greater association or surface exposure of anionic mucus components.

The relatively high total protein EE % indicates efficient retention of the mucus-derived proteinaceous fraction within or in association with the liposomal vesicles. The positive individual coefficients indicate that CH, PC, and DPPC each favored protein encapsulation, with CH showing the strongest effect, followed by DPPC and PC. This may be attributed to the ability of CH to improve bilayer packing and reduce membrane permeability, while DPPC reinforces vesicle rigidity and integrity [58]. However, the negative CH-PC and CH-DPPC interaction terms suggest that excessive lipid packing or bilayer rigidity might reduce the aqueous space or limit accommodation of bulky mucus proteins/glycoproteins. Thus, protein encapsulation required a balance between vesicle stability and sufficient structural flexibility. Allantoin EE % showed wider variation than protein EE %, which is expected because allantoin is a smaller and more water-soluble molecule. CH and PC individually enhanced allantoin retention, with CH exerting the stronger effect, likely through improved membrane packing and reduced leakage as mentioned before. In contrast, DPPC alone negatively affected allantoin EE %, possibly because increased bilayer rigidity reduced the internal aqueous volume available for small hydrophilic molecules [60]. However, the positive CH-DPPC and PC-DPPC interactions indicate that DPPC improved allantoin retention when incorporated within balanced mixed bilayers. The negative higher-order terms suggest that excessive combined lipid effects may over-rigidify the vesicles and reduce allantoin entrapment.

For skin retention, the positive individual coefficients indicate that all three lipids supported protein deposition, with DPPC showing the strongest effect, followed by CH and PC. This suggests that bilayer rigidity and vesicle integrity may promote deposition of the macromolecular mucus fraction [61]. However, the negative binary interactions indicate that excessive combined lipid effects may produce overly rigid or tightly packed vesicles, limiting release and partitioning of proteins into the skin [62]. Similarly, the positive coefficients of CH, PC, and DPPC suggest that each lipid individually supported allantoin deposition, with CH and DPPC showing stronger effects than PC. The negative CH-PC interaction may reflect tighter bilayer packing that restricted release of this small hydrophilic marker as noticed with total protein skin deposition. Overall, these findings demonstrate that optimized lipid ratios are essential to enhance deposition of both macromolecular and low-molecular-weight mucus components.

The optimized formulation maintained the desired nanoscale size, acceptable surface charge, high mucus marker encapsulation, and enhanced cutaneous retention. The low prediction errors confirmed the reliability of the optimization process, while the slightly higher errors observed for zeta potential and skin retention may reflect the inherent variability of surface charge measurements and ex vivo skin assays. Moreover, the higher loading capacity of total protein compared with allantoin is reasonable because the protein value represents the macromolecular mucin/glycoprotein-rich fraction, which may associate more strongly with the liposomal vesicles. In contrast, allantoin is smaller and more water-soluble, making it more likely to remain in the external aqueous phase or be removed during separation.

The layer-specific distribution study demonstrated that Lipo-mucus improved the cutaneous localization of both mucus-derived markers compared with free mucus. Free mucus mainly retained total protein in the stratum corneum, which is expected because the mucin/glycoprotein-rich fraction is large and has limited diffusional mobility [63]. In contrast, Lipo-mucus shifted total protein deposition toward the epidermis and dermis, indicating that liposomal encapsulation did not simply increase surface retention but promoted deeper localization of mucus-derived macromolecules. This improvement likely reflects the ability of phospholipid vesicles to interact with stratum corneum lipids, enhancing skin contact and providing a reservoir for gradual release into viable epidermal layers [64]. Allantoin showed a more pronounced redistribution into deeper skin layers, consistent with its smaller molecular size and greater diffusional capacity compared with total protein. The enhanced epidermal and dermal deposition of both total protein and allantoin suggests that Lipo-mucus improved meaningful cutaneous deposition in skin regions more relevant to anti-aging activity. These results support the role of liposomal encapsulation in enhancing the topical performance of snail mucus.

The slightly lower pH of Lipo-mucus may be attributed to hydration with acetate buffer at pH 5.5. Both values fall within a skin-compatible range, supporting the suitability of the optimized formulation for topical application [65,66]. Rheological analysis showed that Lipo-mucus had lower viscosity than free mucus, suggesting that liposomal incorporation reduced the direct contribution of the native mucin/glycoprotein network to bulk flow behavior. This reduction may decrease stickiness, improve ease of spreading, and enhance cosmetic acceptability [67]. The shear-thinning behavior observed for both formulations is also advantageous for topical application, as the formulation can maintain consistency at rest while spreading more easily under shear during application [68]. Therefore, the viscosity profile and suitable spreadability support the suitability of Lipo-mucus as a more elegant topical anti-aging formulation compared with free mucus. TEM confirmed the formation of spherical to nearly spherical vesicles, in agreement with DLS-based nanoscale characterization. Minor variation in apparent vesicle size may be related to sample drying, negative staining, and possible vesicle flattening during grid preparation.

The absence of visible changes in appearance, color, odor, aggregation, precipitation, creaming, or phase separation indicates acceptable physical stability of Lipo-mucus at room temperature. Although particle size increased slightly during storage, the change was not statistically significant, and the formulation remained within the nanoscale range with acceptable PDI values. This suggests that the vesicular structure and size distribution were largely preserved over the storage period. On the other hand, the non-significant changes in zeta potential further support the colloidal stability of the formulation, while the preservation of total protein and allantoin EE % indicates that both mucus-derived markers remained associated with the liposomal system. In addition, the stability of pH, viscosity, and spreadability suggests that storage did not compromise the topical suitability or cosmetic performance of Lipo-mucus.

The Lipo-mucus formulation demonstrated the greatest overall efficacy, achieving significantly greater wrinkle reduction, skin moisture restoration, and epidermal/dermal thickness on H&E staining than the other treatment groups. This is mechanistically attributable to the phospholipid bilayer architecture of liposomes, which closely resembles the lipid organization of the stratum corneum, facilitating improved dermal penetration, enhanced bioavailability of the encapsulated actives, and sustained release at the target site [50,69,70]. Consistent with this, Masson’s trichrome staining showed an improvement in collagen distribution over the aged control, suggesting that the carrier’s own physicochemical properties, such as improved skin hydration and other barrier functions, make some independent contribution.

Besides the improvement in topical delivery, the increased biological activity of the liposomal formulation was confirmed by coordinated molecular changes associated with the decrease in skin aging. The observed modulation of miR-34a-5p and its downstream regulatory network and restoration of ECM-, angiogenesis-, and senescence-associated markers suggest that the therapeutic benefit of liposomal *H. aspersa* mucus extends beyond improved dermal penetration. These results imply a multi-target mechanism rather than a single pathway involving inhibition of cellular senescence, maintenance of ECM homeostasis, promotion of tissue regeneration and restoration of angiogenic signaling, providing a mechanistic basis for the histological and phenotypic improvements observed in the present study.

At the molecular level, a main finding of this study is the modulation of the miR-34a-5p axis, a specific mature and functional strand of miR-34a. This miR-34a-5p is a p53-regulated microRNA that promotes cellular senescence, cell-cycle arrest, apoptosis, and age-related tissue dysfunction. Its expression increases with aging in dermal fibroblasts and photoaged skin, driving SASP signaling and suppressing tissue regeneration [13,71,72,73,74]. Consistently, aged skin in the present model exhibited pronounced miR-34a-5p upregulation, whereas treatment with snail mucus, particularly in liposomal form, resulted in substantial downregulation, suggesting modulation of upstream stress signaling or direct regulation of microRNA networks to attenuate molecular features associated with skin aging and senescence.

Suppression of miR-34a-5p was accompanied by coordinated restoration of downstream genes governing cellular survival, ECM integrity, and tissue regeneration. Recovery of Met, a receptor tyrosine kinase mediating cell survival, proliferation, and tissue repair, was profoundly suppressed in aged skin and substantially restored following snail mucus treatment. This finding is consistent with an inverse relationship between miR-34a and MET signaling claimed by Simone et al. who demonstrated that miR-34a upregulation in radiation-induced fibrosis suppresses c-Met expression in a murine skin model [75], supporting the mechanistic plausibility that miR-34a-5p downregulation by the mucus contributes to MET reactivation and regeneration in aged skin. Similarly, the marked suppression of Tgif2, a transcriptional repressor of TGF-β/SMAD signaling, in aged skin indicates dysregulated TGF-β activity, that is well recognized as a driver of SASP amplification and ECM imbalance during aging [76,77]. Its restoration following snail mucus treatment may help attenuate SASP-associated fibrosis and ECM abnormalities. This finding is mechanistically in line with evidence that modulation of TGF-β/SMAD signaling pathways can shift senescent dermal fibroblasts toward a more regenerative phenotype [78]. In parallel, the concurrent suppression of Plod1 and Eln in aged skin, encoding lysyl hydroxylase 1 and elastin respectively, reflects ECM fragmentation and loss of elastic fiber organization [79,80], whereas their upregulation following treatment indicates renewed collagen maturation and elastic fiber assembly, consistent with snail mucus-mediated dermal structural regeneration [26,81].

Likewise, Tert, encoding the catalytic subunit of telomerase, was suppressed in aged skin, reflecting impaired telomere maintenance and reduced replicative capacity [82,83]. Elevated telomerase activity promotes telomere elongation, tissue repair, and regenerative capacity, as shown in Tert knock-in mice [84]. Its upregulation by snail mucus treatment suggests partial recovery of telomerase activity and cellular proliferative potential. Although direct evidence linking *H. aspersa* mucus to telomere regulation is lacking, this finding aligns with its broader ECM-restorative and regenerative properties reported in the earlier studies [26,41,85]. Concurrently, aged skin exhibited activation of the p16/CDKN2A–Rb axis, a canonical enforcer of irreversible growth arrest, alongside upregulated Mmp1, indicative of active collagen degradation and ECM remodeling [86,87,88,89]. Snail mucus treatment downregulated both Mmp1 and Cdkn2a expression, reflecting attenuation of ECM degradation and relief from senescence-associated growth arrest, collectively shifting the molecular profile toward a regenerative phenotype [26,49]. Notably, the superior efficacy of the liposomal formulation across this entire gene expression panel reinforces the conclusion that effective transdermal bioavailability is a prerequisite for the full molecular anti-aging activity of *H. aspersa* mucus and positions this formulation as a promising candidate for modulating senescence-associated molecular pathways through miR-34a-5p in aged skin.

Upstream regulatory analysis through X2Kweb situates this interpretation within established aging pathways. Transcription factor enrichment of TP53, E2F1, and SMAD4 aligns with co-elevation of Cdkn2a and suppression of Tgif2 in aged skin [8]. Kinase enrichment identified MAPK family members (p38/MAPK14, ERK1/2, JNK1) that activate AP-1-driven Mmp1 transcription, providing mechanistic context for ECM degradation [90]. DNA damage response kinases ATM and DNAPK link telomere attrition to p16 activation, reinforcing the connection between genomic stress and senescence [91]. Although X2K provides predictive rather than direct evidence of pathway activation, its agreement with experimental data supports multi-target modulation of senescence and ECM pathways by *H. aspersa* mucus.

At the protein level, vascular endothelial growth factor A (VEGFA) and vimentin (VIM) were evaluated as representative markers of tissue regeneration and cytoskeletal remodeling, respectively. The marked decline in VEGFA levels in aged skin reflects impaired angiogenic signaling, contributing to reduced vascularization, compromised tissue perfusion, and diminished regenerative capacity [92,93]. However, restoration of VEGFA following mucus treatment indicates reactivation of angiogenic and regenerative pathways that support endothelial proliferation, tissue repair, and ECM remodeling, which is consistent with the observed improvements in collagen distribution and epidermal and dermal thickness, and the reduced expression of Cdkn2a and Mmp1 [94,95]. Conversely, VIM was markedly elevated in aged skin, reflecting cytoskeletal remodeling and a senescence-associated shift toward a mesenchymal and fibrotic phenotype driven, at least in part, by TGF-β signaling [96,97]. Its suppression after treatment suggests attenuation of fibrosis and restoration of cellular homeostasis [98], consistent with the recovery of Tgif2 expression together with the histological improvement observed in the treated groups. Together, restoration of VEGFA and normalization of VIM support the transcriptomic findings, indicating that liposomal *H. aspersa* mucus promotes angiogenesis while limiting cytoskeletal remodeling and fibrosis. Although miR-34a has been shown to modulate angiogenic signaling, including VEGFA, in some biological contexts [99,100], the regulation of VIM is generally considered indirect and involves broader signaling pathways such as MET, TGF-β, and EMT-related regulators. Hence, the current results should be interpreted as molecular associations and not as evidence of direct mechanistic regulation of VEGFA or VIM by miR-34a-5p.

Overall, the uniformity observed in metabolomic, phenotypic, transcriptomic, and proteomic analyses substantiate the effectiveness of *H. aspersa* mucus as a natural anti-aging topical bioactive ingredient. Nonetheless, Lipo-mucus consistently outperformed the blank liposome across all histological, molecular, and functional endpoints, confirming that the encapsulated snail mucus, rather than the liposomal carrier alone, is the main driver of the observed anti-aging effects.

## 4. Materials and Methods

### 4.1. H. aspersa Mucus Collection and Characterizations

#### 4.1.1. Mucus Collection Protocol

Mucus was collected from *H. aspersa* snails using a gentle, well-controlled procedure designed to obtain sufficient secretion while ensuring the welfare of the snails [101]. All animals were reared under stable laboratory conditions (20–25 °C and humidity above 70%) in ventilated containers and provided with fresh vegetables to maintain good health. During mucus collection, healthy medium-sized snails were lightly stimulated at the pedal region using a sterile fine-tipped metal rod, and the released mucus was collected immediately with a sterile pipette. Throughout the study, animals were handled gently to minimize stress and discomfort, and no euthanasia procedures were performed at any stage. After each session, snails were returned to a moist, food-supplied recovery habitat to restore normal activity. This method provided reliable mucus collection while fully complying with established ethical guidelines for the humane use of invertebrates in scientific research.

#### 4.1.2. Characterization of *H. aspersa* Mucus by LC–ESI–QTOF–MS/MS

Chemical profiling was performed using liquid chromatography electrospray ionization quadrupole time-of-flight tandem mass spectrometry (LC–ESI–QTOF–MS/MS). Chromatographic separation was carried out on an ACQUITY BEH C18 column (1.7 μm) maintained at mean temperature of 30 °C. The mobile phase consisted of solvent A (water containing 0.1% formic acid) and solvent B (acetonitrile containing 0.1% formic acid). A 20 min gradient elution program was applied, starting at 95% A and 5% B, gradually increasing the organic phase to 100% B, followed by re-equilibration to initial conditions.

Mass spectrometric detection was conducted on a Xevo G3 QTof mass spectrometer (Waters Corporation, Milford, MA, USA) equipped with an electrospray ionization source operating in negative ion mode (ESI−). Data were acquired using the MSᴱ data-independent acquisition mode over a mass range of *m*/*z* 100–1200. The low-energy function was operated at a fixed collision energy of 6 V for precursor ion acquisition, while the high-energy function employed a collision energy ramp of 15–45 V to generate fragment ion spectra. The scan time was 0.150 s per function. Raw LC–ESI–QTOF–MS/MS data were processed using UNIFI software (version 3.3.1, Waters Corporation, Milford, MA, USA).

#### 4.1.3. Evaluation of Antioxidant Capacity and Reducing Power of *H. aspersa* Mucus

The antioxidant activity of the mucus was evaluated using the 2,2-diphenyl-1-picrylhydrazyl (DPPH) radical scavenging assay. The *H. aspersa* mucus was tested at concentrations ranging from 50 to 1600 µg/mL. In each well of a 48-well microplate, 100 µL of the mucus was mixed with 100 µL of DPPH solution to achieve a final volume of 200 µL. Then, the plate was incubated in the dark at room temperature, and the absorbance was measured at 517 nm. Radical scavenging activity was expressed as percentage inhibition relative to the control. Trolox was used as the reference antioxidant for the determination of Trolox equivalent antioxidant capacity (TEAC). Percentage inhibition values were converted to Trolox equivalents using a Trolox calibration curve and normalized to the mucus mass present in each well. The concentration required to inhibit 50% of DPPH radicals (IC_50_) was calculated by linear interpolation between the two concentrations closest to 50% inhibition, which were 800 and 1600 µg/mL.

The ferric reducing antioxidant power (FRAP) assay was performed to assess the total antioxidant capacity of the mucus. The assay was conducted in a 48-well microplate by mixing 180 µL of freshly prepared FRAP reagent with 20 µL of either Trolox standard (20–640 µM) or mucus solution at concentrations ranging from 50 to 1600 µg/mL, giving a final volume of 200 µL. After incubation at 37 °C for 4–6 min, absorbance was measured at 593 nm. A Trolox calibration curve was constructed, and the antioxidant capacity of the mucus was expressed as Trolox equivalent antioxidant capacity (TEAC; µmol TE/g mucus).

### 4.2. Liposomal Formulation and Characterization

#### 4.2.1. Preparation of Mucus-Loaded Liposomes

Mucus-loaded liposomes were prepared using the thin-film hydration method [23]. Briefly, cholesterol (CH), phosphatidylcholine (PC) and dipalmitoylphosphatidylcholine (DPPC) were accurately weighed according to the selected D-optimal lipid molar ratios, with a total lipid amount of 30 mg for each formulation. The lipid mixture was dissolved in chloroform:methanol mixture (3 mL). The organic solvent was then removed under reduced pressure using a rotary evaporator (Buchi Rotavapor R-300 Electronic Lift System, Basel, Switzerland). The resulting thin film was kept under vacuum to remove residual solvent, then hydrated with snail mucus solution prepared in acetate buffer (2 mL, pH 5.5) at a concentration of 20 mg/mL. Hydration was performed at 45 °C, slightly above the phase-transition temperature of DPPC, under gentle rotation to allow detachment of the lipid film and formation of multilamellar vesicles. The liposomal dispersion was sequentially extruded through polycarbonate membranes with pore sizes of 400 nm followed by 200 nm (Avanti Research^™^, Alabaster, AL, USA), using 10 passes through each membrane at 45 °C. The prepared mucus-loaded liposomes were stored at 4 °C until further characterization.

#### 4.2.2. Optimization of Mucus-Loaded Liposomes

The composition of mucus-loaded liposomes was optimized using a D-optimal mixture design, in which the molar ratios of the lipid components were varied while keeping the total lipid amount and mucus concentration constant. PC was selected as the principal bilayer-forming phospholipid because of its amphiphilic nature, biocompatibility, and ability to self-assemble into liposomal vesicles [20]. CH was incorporated to modulate bilayer packing and fluidity and to limit membrane permeability and leakage, whereas DPPC, as a saturated phospholipid, was included to enhance bilayer order and vesicle integrity [102,103,104]. The investigated ranges were 55–80 mol% for PC, 15–35 mol% for CH, and 5–20 mol% for DPPC, with the total lipid composition fixed at 100%. These ranges were selected to maintain PC as the predominant structural lipid while providing sufficient variation in CH-mediated membrane packing and DPPC-mediated bilayer rigidity to assess their individual and interactive effects on the critical formulation attributes (Table S1). The selected responses for formulation optimization included particle size, zeta potential, encapsulation efficiency (EE %) based on total protein and allantoin contents as markers of mucus loading, and skin retention of both total protein and allantoin as indicators of cutaneous deposition. The optimized formulation was selected based on minimizing particle size, maximizing the absolute zeta potential value, maximizing mucus marker encapsulation efficiency, and enhancing skin retention.

#### 4.2.3. Measurement of Particle Size and Zeta Potential

The mean particle size, polydispersity index (PDI) and zeta potential of mucus-loaded liposomes were determined by dynamic light scattering using Zetasizer Nano ZS (Malvern Instruments, Southborough, MA, USA). Briefly, liposomal dispersions were diluted 1:100 (*v*/*v*) using deionized water, prior to measurement. Particle size and PDI were measured based on the intensity-weighted distribution, while zeta potential was determined by electrophoretic light scattering at 25 °C [105].

#### 4.2.4. Determination of Mucus Encapsulation Efficiency and Loading Capacity

Non-encapsulated mucus was separated from mucus-loaded liposomes by size-exclusion chromatography using a Sepharose CL-4B column (Sigma-Aldrich, Gillingham, UK) pre-equilibrated with 10 mM phosphate buffer, pH 5.5. An aliquot of liposomal dispersion (0.5 mL) was loaded onto the column and eluted with the same buffer under gravity flow. Fifteen fractions of 0.5 mL were collected per run. Liposome-rich fractions were identified by DLS and phospholipid assay using a Phospholipid Assay Kit (Merck KGaA, Darmstadt, Germany), with absorbance measured at 570 nm and concentrations calculated from a 3–200 µM phospholipid calibration curve. Fractions showing both detectable phospholipid content and measurable DLS signal were considered liposome-rich fractions. The later non-liposomal fractions were analyzed for free total protein and allantoin. Total protein was quantified using the Pierce^™^ BCA Protein Assay Kit (Thermo Scientific, Hemel Hempstead, UK), with absorbance measured at 562 nm and concentrations calculated from a bovine serum albumin calibration curve [105]. Blank liposomes and buffer controls were used to correct for background interference. The BCA assay was employed solely as an indirect analytical approach to estimate the encapsulation of the proteinaceous, mucin-containing fraction of snail mucus within the liposomal system. Total protein content was not considered a measure of mucus biological activity, nor does the BCA assay selectively quantify mucins or individual bioactive glycoproteins. Allantoin was quantified by a validated HPLC-UV method using a Dionex Thermo Scientific system (Thermo Scientific^TM^, Sunnyvale, CA, USA) equipped with a quaternary pump (LPG-3400SD), an autosampler (WPS-3000TSL), a variable-wavelength detector (VWD-3000) and a Nova-Pak C18 column (250 × 4.6 mm, 4 µm; Waters, Helfmann-Park, Eschborn). The mobile phase was acetonitrile: water (80:20, *v*/*v*), delivered isocratically at 1.0 mL/min, with column temperature maintained at 40 °C, injection volume of 10 µL, and detection at 210 nm. The method was linear over 0.5–100 µg/mL (R^2^ = 0.998), with LOD and LOQ values of 0.1 and 0.5 µg/mL, respectively. Precision ranged from 2.11% to 13.97% CV, and mean recovery was 98.14%. The separation profile was first validated using empty liposomes and free mucus solution before calculating indirect encapsulation efficiency according to the following equations:

(7) $$Total\;protein\;ُEE\;(\%)=\frac{Total\;protein\;in\;mucus-free\;quantified\;protein}{Total\;protein\;in\;mucus}\times 100$$

(8) $$Total\;protein\;ُEE\left(\%\right)=\frac{Total\;allantoin\;in\;mucus-free\;quantified\;allantoin}{Total\;allantoin\;in\;mucus}\times 100$$

Loading capacity (LC %) was then calculated relative to the total lipid amount used in the optimized formulation as follows

(9) $$LC\left(\%\right)=\frac{M_{\mathrm{encapsulated}\;\mathrm{marker}}}{M_{\mathrm{total}\;\mathrm{lipid}}}\times 100$$

where M_encapsulated marker_ and M_total lipid_ are the amount of mucus-derived marker either total protein or allantoin associated with the liposomal vesicles and the total mass of lipid used to prepare the formulation, respectively.

#### 4.2.5. Ex Vivo Skin Retention and Cutaneous Distribution Studies

Skin retention of all D-optimal mucus-loaded liposomal formulations was evaluated using excised mouse skin mounted on Franz diffusion cells (Phoenix^™^ RDS; donor volume 1 mL; diffusion area 0.64 cm^2^). After ethical approval, adult male mice were euthanized by intraperitoneal injection of 3% sodium pentobarbital solution at 100 mg/kg body weight [106].

After confirmation of death, dorsal skin was excised, cleaned of subcutaneous fat and connective tissue, visually inspected for integrity, and mounted with the stratum corneum facing the donor chamber. The receptor compartment was filled with 7 mL PBS, pH 7.4, and maintained under continuous magnetic stirring. The diffusion cells were thermostated at 37 ± 0.5 °C. Each formulation was applied at an equivalent mucus dose of 0.2 mL, and free mucus was tested under the same conditions as a comparator. After 24 h, residual formulation was removed using buffer-moistened cotton swabs; the skin surface was washed with saline and gently dried with filter paper [106]. The treated skin area was excised, cut into small pieces, homogenized in 1 mL PBS, pH 7.4, vortex-mixed, and centrifuged at 12,000 rpm for 10 min at 4 °C. The clear supernatant was analyzed for total protein by BCA assay and allantoin by HPLC-UV, as described above. For the optimized formulation, layer-specific skin distribution was further assessed and compared with free mucus. After 24 h exposure under the same Franz diffusion conditions, the remaining formulation was removed and the skin was washed as described above. The stratum corneum was collected by 15 sequential tape strips using adhesive tape strips (Kristall-klar, Tesafilm, Tesa, Norderstedt, Germany) [107]. The remaining skin was separated into epidermis and dermis by immersing it in PBS preheated to 60 °C for 45 s, followed by gentle epidermal peeling using fine forceps [108]. The separated epidermal and dermal fractions were immediately cooled, cut into small pieces, homogenized in PBS, pH 7.4, and centrifuged at 12,000 rpm for 10 min at 4 °C. Supernatants were analyzed for total protein and allantoin. Method recovery was validated for both total retention and layer-specific distribution using blank mouse skin samples spiked with bovine serum albumin and allantoin to confirm acceptable recovery of protein and allantoin, respectively.

#### 4.2.6. pH and Viscosity Measurement of the Optimized Mucus-Loaded Liposome

The optimized mucus-loaded liposomal formulation was assessed for pH and viscosity as indicators of topical suitability and cosmetic acceptability. pH was measured at room temperature using a calibrated digital pH meter (Jenway 3510, Bibby Scientific; Stone, Staffordshire, UK). Viscosity of both mucus and the optimized mucus-loaded liposome was determined at 25 ± 1 °C using a Brookfield DV-III cone (AMETEK Brookfield, Middleboro, MA, USA) and plate rheometer fitted with spindle 52 and controlled by Rheocalc software (Rheocalc 3.3.49 (version 3.3, build 49)). A 0.5 g sample was analyzed at 5, 10, 20, 50, and 100 rpm to assess flow behavior, and the viscosity at 50 rpm was used for comparative reporting.

#### 4.2.7. Spreadability Testing of the Optimized Mucus-Loaded Liposomal Formulation

Spreadability of the optimized mucus-loaded liposomal formulation and free mucus was determined using the parallel-plate method [109]. A fixed amount of each formulation (0.5 g) was placed between two clean glass slides (7.5 × 2.5 cm). A 50 g weight was placed on the upper slide to allow uniform spreading, then removed. The upper slide was then attached to a pulley system and pulled horizontally using a 20 g weight. The time required for the upper slide to move the full slide length was recorded, and spreadability was calculated using the following equation:

(10) $$S=M\times \frac{L}{T}$$

where S represents the spreadability of the formulation, M is the weight attached to the upper slide through the pulley system (20 g), L is the distance traveled by the slide (7.5 cm), and t is the time required for the upper slide to move this distance.

#### 4.2.8. Vesicle Morphology Assessment by Transmission Electron Microscopy

The morphology of the optimized mucus-loaded liposomal formulation was examined by transmission electron microscopy (TEM, JEOL, JEM-1230, Akishima, Tokyo, Japan). A diluted aliquot of the optimized formulation (1:10 *v*/*v*, deionized water) was placed onto a carbon-coated copper grid (300-mesh) and allowed to adsorb for 3 min. Excess sample was removed using filter paper, followed by negative staining with phosphotungstic acid (1 *v*/*v* %) for 1 min. After removal of excess stain, the grid was air-dried at room temperature and examined under TEM at an accelerating voltage of 80 kV.

#### 4.2.9. Stability Study of the Optimized Mucus-Loaded Liposomes

The physicochemical stability of the optimized mucus-loaded liposomal formulation was evaluated during storage at 25 ± 0.5 °C for 3 months. Freshly prepared optimized formulation was transferred into tightly closed glass containers and stored at 25 ± 2 °C, protected from direct light. Samples were withdrawn at predetermined time intervals of 0, 7, 14, 30, 60, and 90 days. At each time point, the formulation was visually inspected for any changes in appearance, color, odor, aggregation, precipitation, creaming, or phase separation. The stability of the formulation was investigated by measuring particle size, PDI, zeta potential, marker EE % and pH, as well as viscosity and spreadability relative to the freshly prepared formulation as mentioned before.

### 4.3. Experimental Animals and Biological Measurements

#### 4.3.1. Experimental Animals and Study Design

The experimental mice were purchased from The Egyptian Organization for Biological Products and Vaccines (VACSERA). Prior to the initiation of the experiment, the mice were acclimatized in acrylic plastic cages under standard laboratory conditions, including a controlled temperature and a 12-h light/12-h dark cycles. Throughout the study, the mice had unrestricted access to tap water and a standard veterinary diet free of contaminants that could potentially interfere with the experimental outcomes.

A total of thirty male mice weighing 30–35 g were randomly allocated into five experimental cohorts (*n* = 6 per group). The first group comprised young mice (2 months old) and served as the standard control (young). The remaining four groups comprised elderly mice (24 months old), with one group labeled as the elderly control (control aged). The remaining three aged mice groups received the following treatments: liposomal formulation without snail mucus (Lipo), free snail mucus (free mucus), and liposomal snail mucus formulation (Lipo-mucus). Prior to the topical treatment, the dorsal skin of all animals was carefully shaved. The assigned formulations were applied topically at a dose of 0.2 mL/day/mouse [110] for one month.

At the end of the experiment, dorsal skin appearance was documented using standardized digital photography, followed by wrinkle scoring and evaluation of skin moisture content. Subsequently, animals were euthanized by intraperitoneal injection of 3% sodium pentobarbital solution at 100 mg/kg body weight, and full-thickness dorsal skin samples were removed and divided for further analyses. Some of these samples were preserved in formalin for histopathological examination, while the residual samples were prepared for molecular and biochemical studies.

#### 4.3.2. Grading of Wrinkles

Wrinkle formation was assessed using a modified technique outlined by Bissett et al. [111]. Upon completion of the experiment, standardized photographs were taken of the dorsal skin of the mice. The extent of wrinkle formation was evaluated utilizing a magnifying lens based on a predetermined grading scale (Table 2). Two impartial evaluators, unaware of the treatment groups, assessed the wrinkles individually.

#### 4.3.3. Skin Moisture Content

Skin moisture content was determined using a gravimetric method immediately after samples’ collection. Briefly, approximately 1.5 g of skin tissue was accurately weighed (wet weight) and then dried overnight in a standard laboratory oven at 80 °C. After drying, samples were reweighed to obtain the dry weight [112]. The percentage of moisture content was calculated using the following equation:

(11) $$\%\mathrm{Moisture}\;\mathrm{Content}=\frac{\mathrm{Wet}\mathrm{Weight}-\mathrm{Dry}\mathrm{Weight}}{\mathrm{Wet}\mathrm{Weight}}\times 100$$

#### 4.3.4. Histological Examination

Following fixation in 10% neutral formol and paraffin embedding, the dorsal skin sections of 4–5 μm thickness from the experimental mice underwent the standard histological staining with H&E for structural examination and Masson’s trichrome staining for differentiating the collagen fiber distribution at the dermal matrix. All stained slices were observed under a light Olympus BX-51 microscope (Tokyo, Japan, ×200 magnification), and representative images had been photographed by an Olympus digital camera. The thickness of epidermal and dermal layers (measured in μm) as well as collagen deposition (presented as a percentage of blue-stained collagen area relative to the total field area) were assessed by Image J software (ImageJ, v1.53 k) in non-overlapping randomly selected fields from each group (*n* = 5) [113,114].

#### 4.3.5. Determination of miR-34a-5p

MiRNA-34a-5p expression was quantified using a TaqMan^™^ MicroRNA Assay specific for mmu-miR-34a (Assay ID: 000426). Reverse transcription was performed using the TaqMan^™^ MicroRNA Reverse Transcription Kit (Cat. No. 4366596), followed by quantitative real-time PCR using TaqMan^™^ Universal PCR Master Mix II, no UNG (Cat. No. 4440040) on StepOnePlus^™^ Real-Time PCR system (Thermo Fisher Scientific, Waltham, MA, USA). snoRNA202 (Assay ID: 001232) was used as the endogenous control. All kits and assays were purchased from Thermo Fisher Scientific (Waltham, MA, USA) and used according to the manufacturer’s instructions. Relative expression levels were calculated using the 2^^−ΔΔCt^ method.

#### 4.3.6. In Silico Identification of miR-34a-5p Target Genes and Upstream Regulatory Networks

The target genes for mmu-miR-34a-5p were detected in https://mirdb.org/ [114]. Only genes with a target score ≥ 80 were chosen as an input to X2Kweb (https://maayanlab.cloud/X2K/, accessed on 21 March 2026) to infer upstream regulatory networks potentially associated with this targetome. X2Kweb integrates transcription factor enrichment analysis (TFEA) and kinase enrichment analysis (KEA) to spotlight candidate transcription factors and kinases that are statistically enriched upstream of the submitted genes. It is worth mentioning that a transcription factor could exist more than once in the ranked list if it is supported by multiple independent ChEA/ENCODE experiments, each yielding a distinct overlapping target-gene set and associated *p*-value. Analyses were performed using default platform settings, and enrichment significance was reported using the default *p*-values of the platform [115].

#### 4.3.7. Selection of Experimentally Detected Genes

From the miRDB-derived miR-34a-5p target list, some genes were selected based on their biological domains related to skin aging, including ECM regulation (met proto-oncogene (Met), TGFB-induced factor homeobox 2 (Tgif2), and procollagen-lysine, 2-oxoglutarate 5-dioxygenase 1 (Plod1)) as well as collagen degradation (MMPs). Although Mmp25 was identified as a predicted miR-34a-5p target, Mmp1 was selected for experimental evaluation due to its direct and well-documented role as the principal interstitial collagenase.

However, the experimental work was not limited to the predicted miR-34a targets; additional genes were also selected to comprehensively evaluate the aging phenotype in skin. These included cyclin-dependent kinase inhibitor 2A (Cdkn2a), which is a cellular senescence marker, elastin (Eln), which reflects elastic fiber integrity, and telomerase reverse transcriptase (Tert), which maintains and extends telomeres.

#### 4.3.8. Determination of Met, Tgif2, Plod1, Mmp1, Cdkn2a, Eln, and Tert by qRT-PCR

Total RNA was extracted from mouse dorsal skin tissue using the TRIzol Reagent (Cat # 15596026) according to the manufacturer’s instructions. RNA concentration and purity were assessed using a NanoDrop spectrophotometer (Thermo Fisher Scientific, Waltham, MA, USA). Complementary DNA (cDNA) was synthesized from 1 µg of total RNA using the SuperScript^™^ IV First-Strand Synthesis System (Cat # 18091200). Gene expression levels were quantified by PowerUp^™^ SYBR^™^ Green Master Mix for qPCR (Cat # A25742) on StepOnePlus^™^ Real-Time PCR system (Thermo Fisher Scientific, Waltham, MA, USA). All kits and assays were purchased from Thermo Fisher Scientific (Waltham, MA, USA) and used according to the manufacturer’s instructions. Thermal cycling conditions were 95 °C for 2 min, followed by 40 cycles of 95 °C for 3 s and 60 °C for 30 s. β-actin was used as the housekeeping gene for normalization. Relative gene expression was calculated using the 2^^−ΔΔCt^ method. Primer sequences for all genes are listed in Table S17.

#### 4.3.9. Determination of VEGFA and Vimentin by ELISA

Protein level of vascular endothelial growth factor A (VEGFA) and vimentin (VIM) were measured using commercial ELISA kits following the manufacturer’s protocols. The assays, supplied by Elabscience (Wuhan, China), had Cat # ELK1293 and ELK3731 for mouse VEGFA and vimentin, respectively. Tissue homogenates were processed for analysis, and the absorbance was read with a microplate reader at 450 nm.

### 4.4. Statistical Analysis

Data analysis was conducted using IBM SPSS Statistics 20 (IBM Corp., Armonk, NY, USA). Quantitative data were represented as mean ± standard deviation (SD), while ordinal data were expressed as median with interquartile range (IQR). Data normality was assessed using the Shapiro–Wilk test and visual inspection of normal Q–Q plots, while homogeneity of variances was evaluated using Levene’s test. Normally distributed data with equal variances were analyzed using one-way ANOVA followed by Tukey’s post hoc test. When the assumption of equal variances was violated, Welch’s ANOVA followed by the Games–Howell post hoc test was performed. Given the ordinal nature of the wrinkle-grading scale, the Kruskal–Wallis test was used, followed by Dunn–Bonferroni post hoc correction. A *p*-value < 0.05 was deemed to be statistically significant.

The sample size was assumed based on the primary outcome (miR-34a-5p expression) using a two-sided significance level of 0.05, 80% statistical power, and an assumed large standardized effect size (Cohen’s d = 1.8), which is commonly considered appropriate for preclinical animal studies. This analysis indicated that a minimum of six mice per group was required; six mice were accordingly included in each experimental group. Following completion of the study, a post hoc power analysis based on miR-34a-5p expression demonstrated that the achieved statistical power exceeded 99.9% for the overall one-way ANOVA (Cohen’s f = 4.43), confirming that the sample size was more than adequate to detect between-group differences.

## 5. Limitations of the Study

The complex and variable composition of snail mucus, together with the lack of a comprehensive reference database for its constituents, limits identification of the specific bioactive compounds responsible for the observed anti-aging effects. Standardized public databases and fractionation-guided bioassays are needed to identify the compounds specifically responsible for miR-34a-5p modulation. In addition, although this study demonstrated simultaneous modulation of miR-34a-5p, VEGFA and VIM, the direct causality between them was not assessed. Therefore, more functional and translational studies are recommended, alongside other ECM markers and downstream effectors. The study design also represented the minimum structure needed to distinguish the effects of mucus encapsulation from the carrier alone, without a dose–response arm, an established positive/reference comparator, or multiple treatment time points. Consequently, future work should incorporate these elements to characterize dose-dependency and durability of the effect. Direct tissue-level oxidative stress markers would strengthen future mechanistic validation of the DPPH/FRAP-based antioxidant findings. Finally, these results were obtained in a mouse model, but further validation in translational models such as ex vivo/human skin, followed by clinical studies, is warranted to confirm the relevance of these findings for human application and to clarify the precise molecular interplay between snail mucus components and microRNA regulatory networks.

## 6. Conclusions

**Figure 10:**
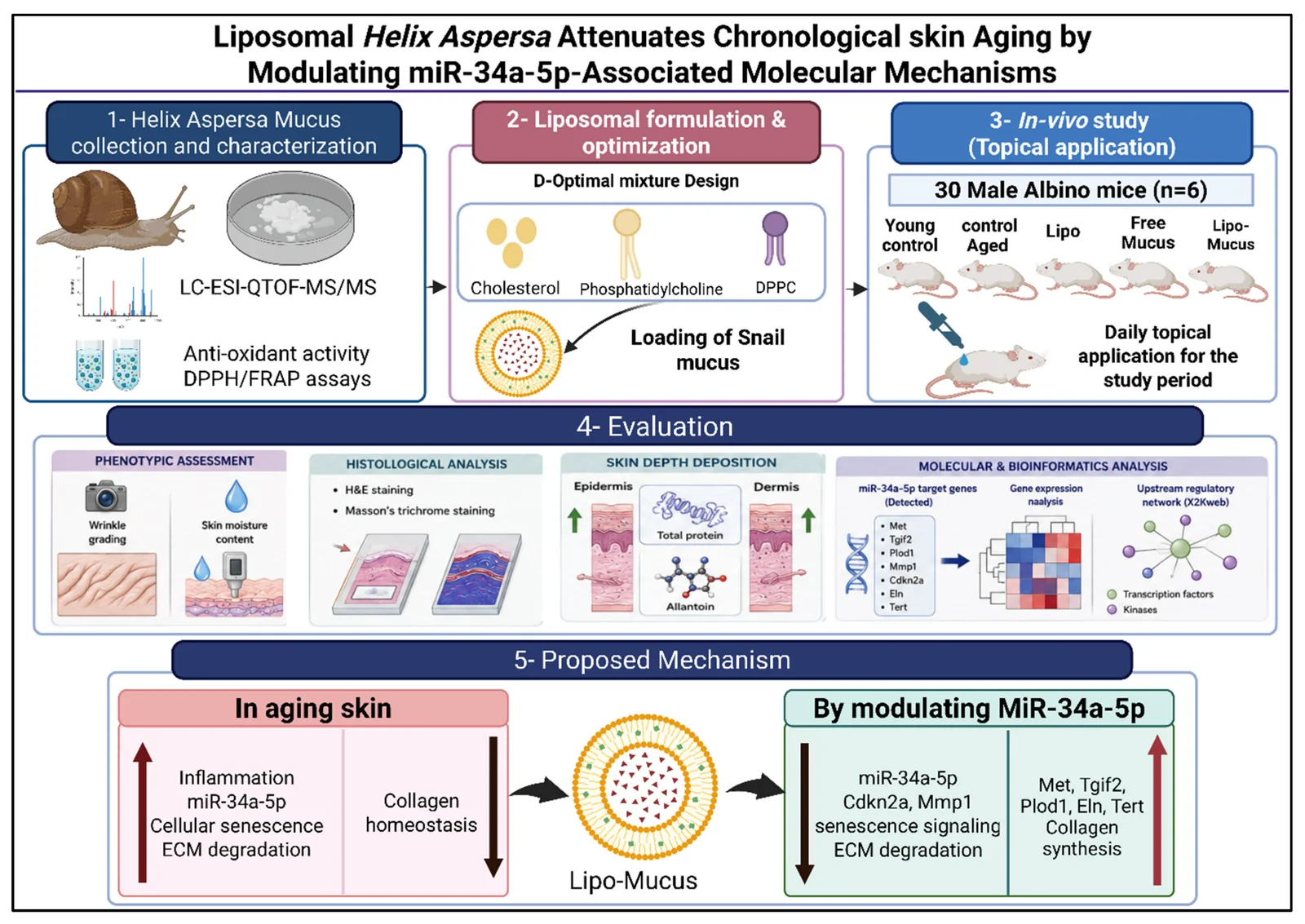
Schematic representation of the proposed molecular mechanisms by which liposomal *H. aspersa* mucus attenuates chronological skin aging through modulation of miR-34a-5p-associated signaling pathways.

*H. aspersa* mucus has substantial anti-aging effects through coordinated modulation of miR-34a-5p-associated molecular pathways. The downregulation of miR-34a-5p correlated with the restoration of key signaling cascades that regulate cellular survival, ECM integrity, angiogenesis, and telomeric maintenance, while concurrently inhibiting senescence- and matrix-degrading factors. These effects were more pronounced with the liposomal formulation. The optimized formulation, Lipo-mucus, demonstrated that liposomal encapsulation can improve the topical performance of *H. aspersa* mucus by enhancing mucus-marker loading, cutaneous deposition, and cosmetic acceptability compared with free mucus. The improved localization of total protein and allantoin within epidermal and dermal layers supports the potential of this system for anti-aging skincare applications. In addition, the formulation maintained its physicochemical properties, marker retention, and organoleptic characteristics over 90 days, indicating its promise as a stable and cosmetically elegant snail mucus-based nanocosmeceutical platform (Figure 10).

## Figures and Tables

**Table 1:** Physicochemical and cosmetic characterization of the optimized Lipo-mucus.

LC (%)		pH ^c,f^		Viscosity (cP) ^d,f^		Spreadability (g.cm/s) ^e,f^	
Total Protein ^a,f^	Allantoin ^b,f^	Mucus	Lipo-Mucus	Mucus	Lipo-Mucus	Mucus	Lipo-Mucus
8.79 ± 2.76	6.25 ± 1.96	6.89 ± 1.24	5.88 ± 0.98	248.33 ± 27.18	159 ± 11.86	7.81 ± 1.25	13.65 ± 2.03

^a^ Total protein LC % was calculated as a ratio of the entrapped total protein to the mass of total lipid. ^b^ Allantoin LC % was calculated as a ratio of the entrapped allantoin to the mass of total lipid. ^c^ pH was measured at room temperature. ^d^ Viscosity was determined at 25 ± 1 °C using a Brookfield DV-III cone and plate rheometer fitted with spindle 52. ^e^ Spreadability was determined using the parallel-plate method. ^f^ Expressed as mean ± SD (*n* = 3).

**Table 2:** Grading scale for wrinkle assessment.

Grade	Description of Wrinkling
**0**	No visible wrinkles
**2**	Few shallow wrinkles across the dorsal skin
**4**	Moderate number of coarse wrinkles across the dorsal skin
**6**	Several deep and coarse wrinkles across the dorsal skin
**8**	Numerous deep and long wrinkles across the dorsal skin

## Data Availability

The original contributions presented in this study are included in the article/Supplementary Materials. Further inquiries can be directed to the corresponding authors.

## Supplementary Materials

The following supporting information can be downloaded at https://www.mdpi.com/article/10.3390/ijms27177729/s1.
