# Supplementary material for: Liposomal Helix aspersa Snail Mucus: A Biomacromolecular Platform for Attenuating Chronological Skin Aging Through Modulation of miR-34a-Associated Senescence and Extracellular Matrix Remodeling
Source: Int J Mol Sci. 2026 Aug 28;27(17):7729. doi: 10.3390/ijms27177729 (PMC13566471; doi:10.3390/ijms27177729)
Supplement: Supplementary file 1 [file ijms-27-07729-s001.zip › ijms-4456608-supplementary.pdf]

Table (S1): Independent factors and response variables used in the D-optimal optimization of mucus-loaded liposomes.

Factors	Levels (mole %)	
	Low	High
A: CH [a]	15	35
B: PC [b]	55	80
C: DPPC [c]	5	20
Responses	Constrains	
Y1: Particle size (nm)	Minimize	
Y2: Zeta potential (mV)	Maximize the absolute value	
Y3: Total protein EE (%)	Maximize	
Y4: Allantoin EE (%)	Maximize	
Y5: Total protein skin retention (µg/cm^2)	Maximize	
Y6: Total allantoin skin retention (µg/cm^2)	Maximize	

[a] is Cholesterol, [b] Phosphatidylcholine, [c] is Dipalmitoylphosphatidylcholine

Table (S2): Experimental design matrix of tested variables and the related responses.

Run	Lipid mole %			Responses					
	**A: CH**	**B: PC**	**C: DPPC**	**Particle size (nm) [a, g]**	**Zeta potential (mV) [b, g]**	**Total protein EE (%) [c, g]**	**Allantoin EE (%) [d, g]**	**Total protein skin retention (µg/ cm^2) [e, g]**	**Total allantoin skin retention (µg/ cm^2) [f, g]**
1	15	72.43	12.57	205.03±24.12	-7.15±1.11	76.5±3.11	51.6±9.11	26.23±3.65	7.17±0.58
2	15	65	20	201.99±15.69	-17.83±3.65	80.68±5.63	51.36±6.47	30.33±4.12	8.23±0.98
3	15	65	20	198.30±14.36	-16.82±2.59	78.98±4.69	48.11±3.69	27.34±2.36	9.16±1.45
4	35	55	10	192.93±10.65	-13.2±3.25	82.68±8.65	67.36±5.78	40.54±3.54	8.55±1.58
5	25	55	20	202.38±22.04	-13.8±1.98	77.1±10.64	64.15±6.48	40±4.12	10.1±2.04
6	21.69	61.27	17.04	206.86±13.25	-22.6±2.36	75.29±5.78	71.58±3.96	29.12±3.78	6.5±1.03
7	35	55	10	193.11±5.96	-13.34±3.14	83.47±12.35	69.87±5.89	41.83±3.65	9.75±2.31
8	15	80	5	203.11±11.32	-13.63±1.45	74.18±8.96	59.74±4.15	36.52±4.52	5.13±0.87
9	15	80	5	205.18±26.35	-12.64±1.65	75.71±14.36	63.04±3.29	38.66±4.69	4.21±0.26
10	28.36	59.79	11.85	203.91±12.03	-12.52±1.85	76.89±14.65	55.49±8.41	31.91±3.68	8.08±1.06
11	21.04	73.46	5.5	211.79±14.63	-8.36±1.03	75.41±6.58	77.36±6.65	32.96±4.69	1.83±0.21
12	33.67	61.33	5	201.62±10.36	-20.19±1.98	81.91±10.63	55.07±6.14	39.8±3.65	2.86±0.36

13	25	55	20	200±14.65	-13.44±1.65	77.85±12.36	6017±9.11	38.12±5.69	10.22±1.54
14	33.67	61.33	5	201.5±13.98	-19.17±1.47	82.87±10.65	55.11±8.98	41.03±4.69	3.14±0.54
15	27.82	67.18	5	211±10.45	-12.93±1.63	77.77±9.36	65.06±6.98	34.08±2.69	1.11±0.31
16	22.45	66.37	11.18	210.98±10.96	-8.21±1.31	75.11±14.69	58.76±10.33	26.55±3.25	5.85±0.87

[a] Particle size was measured by DLS
[b] Zeta potential was measured by electrophoretic light scattering
[c] Total protein EE % was quantified indirectly by BCA
[d] Allantoin EE % was quantified indirectly by HPLC
[e] Total protein skin retention was determined in rat tissue homogenate after 24 h using BCA
[f] Allantoin skin retention was determined in rat tissue homogenate after 24 h using HPLC
[g] Expressed as mean ± SD (n=3)

Table (S3): Model summary statistics for particle size (Y1).

Source	Std. Dev.	R^2	Adjusted R^2	Predicted R^2	PRESS
Linear	6.67	0.2305	0.1121	-0.2492	938.53
**Quadratic [a]**	**0.9929**	**0.9869**	**0.9803**	**0.9590**	**30.78**
Special Cubic	1.01	0.9879	0.9798	0.9550	33.78
Cubic	1.21	0.9882	0.9706	0.6227	283.49
Special Quartic	1.14	0.9879	0.9740	0.0471	715.93
Quartic	1.29	0.9889	0.9666		*

[a] Adequate precision is 24.54 and coefficient of variation (CV%) equals 0.62
[*] Aliased

Table (S4): Model summary statistics for zeta potential (Y2).

Source	Std. Dev.	R^2	Adjusted R^2	Predicted R^2	PRESS
Linear	4.39	0.1084	-0.0288	-0.2715	356.57
Quadratic	3.94	0.4477	0.1715	-0.0892	305.44
Special Cubic	2.50	0.8002	0.6670	0.2457	211.54
**Cubic [a]**	**0.5165**	**0.9943**	**0.9857**	**0.9669**	**9.28**
Special Quartic	0.6169	0.9905	0.9796	-0.6624	466.19
Quartic	0.5648	0.9943	0.9829		*

[a] Adequate precision is 37.85 and coefficient of variation (CV%) equals 3.66
[*] Aliased

Table (S5): Model summary statistics for total protein EE% (Y3).

Source	Std. Dev.	R^2	Adjusted R^2	Predicted R^2	PRESS
Linear	2.29	0.5098	0.4344	0.2589	103.27
**Quadratic**	**0.6793**	**0.9669**	**0.9503**	**0.9049**	**13.25**
Special Cubic	0.6959	0.9687	0.9479	0.8937	14.81
Cubic	0.7821	0.9737	0.9342	0.8961	14.48
Special Quartic	0.7776	0.9696	0.9349	-1.3499	327.42
Quartic	0.8567	0.9737	0.9210		*

[a] Adequate precision is 19.29 and coefficient of variation (CV%) equals 0.86
[*] Aliased

Table (S6): Model summary statistics for allantoin EE% (Y4).

Source	Std. Dev.	R^2	Adjusted R^2	Predicted R^2	PRESS
Linear	8.19	0.1145	-0.0217	-0.2941	1273.31
Quadratic	7.44	0.4375	0.1563	-0.1979	1178.69
Special Cubic	7.82	0.4405	0.0675	-0.7540	1725.85
**Cubic**	**1.91**	**0.9778**	**0.9446**	**0.9005**	**97.89**
Special Quartic	6.80	0.6712	0.2954	-198.4324	1.962E+05
Quartic	2.09	0.9778	0.9335		*

[a] Adequate precision is 18.37 and coefficient of variation (CV%) equals 3.13
[*] Aliased

Table (S7): Model summary statistics for total protein skin retention (Y5).

Source	Std. Dev.	R^2	Adjusted R^2	Predicted R^2	PRESS
Linear	4.49	0.4356	0.3488	0.1378	399.69
**Quadratic**	**1.07**	**0.9752**	**0.9628**	**0.9254**	**34.59**
Special Cubic	1.13	0.9754	0.9590	0.9161	38.90
Cubic	1.33	0.9771	0.9428	0.3678	293.04
Special Quartic	1.28	0.9754	0.9473	0.8340	76.96
Quartic	1.42	0.9782	0.9345		*

[a] Adequate precision is 24.06 and coefficient of variation (CV%) equals 3.09
[*] Aliased

Table (S8): Model summary statistics for allantoin skin retention (Y6).

Source	Std. Dev.	R^2	Adjusted R^2	Predicted R^2	PRESS
Linear	1.82	0.6861	0.6378	0.5167	66.33
**Quadratic**	**0.7288**	**0.9613**	**0.9420**	**0.9155**	**11.59**
Special Cubic	0.7671	0.9614	0.9357	0.8813	16.29
Cubic	0.5362	0.9874	0.9686	0.5615	60.18
**Special Quartic**	**0.4944**	**0.9875**	**0.9733**	**0.4283**	**78.47**
Quartic	0.5696	0.9882	0.9645		*

[a] Adequate precision is 19.9 and coefficient of variation (CV%) equals 11.44
[*] Aliased

Table (S9): ANOVA of the obtained data from D-optimal design for the particle size of mucus loaded liposomes and associated p-values.

Source	Sum of Squares	Df [a]	Mean Square	F-value	p-value [b]
**Model**	448.58	5	89.72	55.74	< 0.0001
[(1)]Linear Mixture	137.84	2	68.92	42.82	< 0.0001
AB	310.07	1	310.07	192.65	< 0.0001
AC	55.88	1	55.88	34.72	0.0002
BC	8.65	1	8.65	5.37	0.0429
**Residual**	16.09	10	1.61		
Lack of Fit	4.51	5	0.9017	0.3891	0.8383
Pure Error	11.59	5	2.32		
**Cor Total**	464.67	15			

[a] Degree of freedom
[b] *$p < 0.05$

Table (S10): ANOVA of the obtained data from D-optimal design for the zeta potential of mucus loaded liposomes and associated p-values.

Source	Sum of Squares	df	Mean Square	F-value	p-value
**Model**	278.82	9	30.98	116.12	< 0.0001
(1)Linear Mixture	30.39	2	15.19	56.95	0.0001
AB	13.23	1	13.23	49.58	0.0004
AC	19.67	1	19.67	73.74	0.0001
BC	28.14	1	28.14	105.46	< 0.0001
ABC	15.57	1	15.57	58.38	0.0003
AB(A-B)	0.2363	1	0.2363	0.8857	0.3830
AC(A-C)	49.92	1	49.92	187.11	< 0.0001
BC(B-C)	47.21	1	47.21	176.96	< 0.0001
**Residual**	1.60	6	0.2668		
Lack of Fit	0.0059	1	0.0059	0.0184	0.8974
Pure Error	1.59	5	0.3190		
**Cor Total**	280.42	15			

[a] Degree of freedom
[b] *p< 0.05

Table (S11): ANOVA of the obtained data from D-optimal design for the total protein EE% of mucus loaded liposomes and associated p-values.

Source	Sum of Squares	df	Mean Square	F-value	p-value
**Model**	134.72	5	26.94	58.40	< 0.0001
(1)Linear Mixture	71.04	2	35.52	76.98	< 0.0001
AB	30.06	1	30.06	65.16	< 0.0001
AC	33.78	1	33.78	73.22	< 0.0001
BC	2.09	1	2.09	4.52	0.0593
**Residual**	4.61	10	0.4614		
Lack of Fit	0.9443	5	0.1889	0.2573	0.9187

Pure Error	3.67	5	0.7339		
**Cor Total**	139.34	15			

[a] Degree of freedom
[b] *p< 0.05

Table (S12): ANOVA of the obtained data from D-optimal design for the allantoin EE% of mucus loaded liposomes and associated p-values.

**Source**	**Sum of Squares**	**df**	**Mean Square**	**F-value**	**p-value**
**Model**	962.12	9	106.90	29.40	0.0003
(1)Linear Mixture	112.68	2	56.34	15.49	0.0043
AB	18.87	1	18.87	5.19	0.0630
AC	96.56	1	96.56	26.55	0.0021
BC	110.09	1	110.09	30.27	0.0015
ABC	72.05	1	72.05	19.81	0.0043
AB(A-B)	323.61	1	323.61	88.99	< 0.0001
AC(A-C)	173.40	1	173.40	47.68	0.0005
BC(B-C)	133.72	1	133.72	36.77	0.0009
**Residual**	21.82	6	3.64		
Lack of Fit	0.0222	1	0.0222	0.0051	0.9459
Pure Error	21.80	5	4.36		
**Cor Total**	983.94	15			

[a] Degree of freedom
[b] *p< 0.05

Table (S13): ANOVA of the obtained data from D-optimal design for the total protein skin retention of mucus loaded liposomes and associated p-values.

**Source**	**Sum of Squares**	**df**	**Mean Square**	**F-value**	**p-value**
**Model**	452.08	5	90.42	78.74	< 0.0001
(1)Linear Mixture	201.93	2	100.96	87.93	< 0.0001

AB	88.94	1	88.94	77.45	< 0.0001
AC	71.47	1	71.47	62.25	< 0.0001
BC	155.88	1	155.88	135.75	< 0.0001
**Residual**	11.48	10	1.15		
Lack of Fit	1.37	5	0.2734	0.1351	0.9767
Pure Error	10.12	5	2.02		
**Cor Total**	463.56	15			

[a] Degree of freedom
[b] *p< 0.05

Table (S14): ANOVA of the obtained data from D-optimal design for the allantoin skin retention of mucus loaded liposomes and associated p-values.

**Source**	**Sum of Squares**	**df**	**Mean Square**	**F-value**	**p-value**
**Model**	131.93	5	26.39	49.68	< 0.0001
(1)Linear Mixture	94.16	2	47.08	88.65	< 0.0001
AB	22.94	1	22.94	43.19	< 0.0001
AC	2.30	1	2.30	4.33	0.0642
BC	2.10	1	2.10	3.96	0.0747
**Residual**	5.31	10	0.5311		
Lack of Fit	3.69	5	0.7378	2.27	0.1941
Pure Error	1.62	5	0.3244		
**Cor Total**	137.24	15			

[a] Degree of freedom
[b] *p< 0.05

Table (S15): The experimental and predicted of the optimized Lipo-mucus

**Response**	**Predicted**	**Experimental**	**Predicted error (%)**
Particle size (nm) [a. g]	193.01	201.65±7.89	4.48
Zeta potential (mV) [b. g]	-13.28	-15.11±2.69	13.78
Total protein EE (%) [c. g]	82.85	85.96± 5.89	3.75
Allantoin EE (%) [d. g]	68.6	70.69±6.98	3.05
Total protein skin retention (μg/ cm^2) [e. g]	41.42	46.89±4.69	13.21

Allantoin skin retention (μg/ cm^2) [f, g]	9.29	10.69±2.47	15.07

[a] Particle size was measured by DLS
[b] Zeta potential was measured by electrophoretic light scattering
[c] Total protein EE % was quantified indirectly by BCA
[d] Allantoin EE % was quantified indirectly by HPLC
[e] Total protein skin retention was determined in rat tissue homogenate after 24 h using BCA
[f] Allantoin skin retention was determined in rat tissue homogenate after 24 h using HPLC
[g] Expressed as mean ± SD (n=3)

Table (S16): Stability assessment of Lipo-mucus after storage at 25 °C for 90 days

**Parameters**	**Storage at 25 °C (days)**					
	**0**	**7**	**14**	**30**	**60**	**90**
Particle size (nm) [a, h]	201.65±7.89	215.32±10.36	207.36±9.87	229.65±16.36	226.36±18.65	232.32±20.36
PDI [a, h]	0.25±0.033	0.23±0.04	0.29±0.01	0.3±0.04	0.28±0.01	0.29±0.02
Zeta potential (mV) [b, h]	-15.11±2.69	-12.36±1.85	-16.98±3.65	-14.63±2.04	-16.65±1.65	-14.96±1.58
Total protein EE (%) [c, h]	85.96± 5.89	86.96±3.58	82.04±4.98	85.07±7.25	81.36±4.78	81.02±3.65
Allantoin EE (%) [d, h]	70.69±6.98	72.06±3.28	69.65±4.65	70.36±10.36	67.36±6.05	68.39±4.65
pH [e, h]	5.88±0.98	6.01±1.21	5.84±1.87	5.32±0.78	5.87±0.69	5.57±1.06
Viscosity (cP) [f, h]	159±14.53	165.32±10.36	150.14±15.39	130.36±25.71	128.69±13.05	140.69±20.36
Spreadability (g.cm/s) [g, h]	13.65± 2.03	14.32±1.65	12.05±2.07	13.98±1.45	14.65±2.36	13.45±1.85

[a] Particle size was measured by DLS
[b] Zeta potential was measured by electrophoretic light scattering
[c] Total protein EE % was quantified indirectly by BCA
[d] Allantoin EE % was quantified indirectly by HPLC
[e] pH is measured at room temperature
[f] Viscosity was determined at 25 ± 1 °C using a Brookfield DV-III cone and plate rheometer fitted with spindle 52
[g] Spreadability was determined using the parallel-plate method
[h] Expressed as mean ± SD (n=3)

Table (S17): Primer sequences used for qRT-PCR.

Gene	Forward Primer	Reverse Primer
Met	GTTCTGCTTGGCAACGAGAGCT	GGAGAATGCACTGTATTGCGTCG
Tgif2	CCTCAGAGCAGGAGAAGCTAAG	GGTCTTTGCCATCCTTCCGAAG
Plod1	CTGCCGAATCTTCCAGAACCTG	CCATTGCCATGAACGACCACTG
Mmp1	ACTACAACTGACAACCCAAGAA	AGTGTCTCTTAGCTGGGCAC
Cdkn2a	TGTTGAGGCTAGAGAGGATCTTG	CGAATCTGCACCGTAGTTGAGC
Eln	TCCCGGTGGAGTCTATTATC	CACCTCCAAGTCCTCCAG
Tert	CTCTCTGCTGCGCAGCCGATAC	CCTCGTTAAGCAGCTCAAAG
β-actin	GGCTGTATTCCCCTCCATCG	CCAGTTGGTAACAATGCCATGT

Met: met proto-oncogene, Tgif2: TGFB-induced factor homeobox 2, Plod1: procollagen-lysine, 2-oxoglutarate 5-dioxygenase 1, Mmp1: matrix metallopeptidase, Cdkn2a: cyclin-dependent kinase inhibitor 2A, Eln: elastin, Tert: telomerase reverse transcriptase.
